# Rapid enhancement of multiple ecosystem services following the restoration of a coastal foundation species

**DOI:** 10.1002/eap.2466

**Published:** 2021-10-25

**Authors:** Kathryn M. Beheshti, Susan L. Williams, Katharyn E. Boyer, Charlie Endris, Annakate Clemons, Tracy Grimes, Kerstin Wasson, Brent B. Hughes

**Affiliations:** ^1^ Department of Ecology and Evolutionary Biology University of California, Santa Cruz Santa Cruz California 95060 USA; ^2^ Department of Ecology and Evolutionary Biology University of California, Davis Davis California 95616 USA; ^3^ Estuary & Ocean Science Center San Francisco State University Tiburon California 94920 USA; ^4^ Moss Landing Marine Laboratories Moss Landing California 95039 USA; ^5^ Department of Ecology San Diego State University San Diego California 92182 USA; ^6^ Elkhorn Slough National Estuarine Research Reserve Royal Oaks California 95076 USA; ^7^ Department of Biology Sonoma State University Rohnert Park California 94928 USA

**Keywords:** carbon stocks, community ecology, ecosystem services, eelgrass, experimental restoration, multifunctionality, nursery habitat, species richness, water quality, *Zostera marina*

## Abstract

The global decline of marine foundation species (kelp forests, mangroves, salt marshes, and seagrasses) has contributed to the degradation of the coastal zone and threatens the loss of critical ecosystem services and functions. Restoration of marine foundation species has had variable success, especially for seagrasses, where a majority of restoration efforts have failed. While most seagrass restorations track structural attributes over time, rarely do restorations assess the suite of ecological functions that may be affected by restoration. Here we report on the results of two small‐scale experimental seagrass restoration efforts in a central California estuary where we transplanted 117 0.25‐m^2^ plots (2,340 shoots) of the seagrass species *Zostera marina*. We quantified restoration success relative to persistent reference beds, and in comparison to unrestored, unvegetated areas. Within three years, our restored plots expanded ~8,500%, from a total initial area of 29 to 2,513 m^2^. The restored beds rapidly began to resemble the reference beds in (1) seagrass structural attributes (canopy height, shoot density, biomass), (2) ecological functions (macrofaunal species richness and abundance, epifaunal species richness, nursery function), and (3) biogeochemical functions (modulation of water quality). We also developed a multifunctionality index to assess cumulative functional performance, which revealed restored plots are intermediate between reference and unvegetated habitats, illustrating how rapidly multiple functions recovered over a short time period. Our comprehensive study is one of few published studies to quantify how seagrass restoration can enhance both biological and biogeochemical functions. Our study serves as a model for quantifying ecosystem services associated with the restoration of a foundation species and demonstrates the potential for rapid functional recovery that can be achieved through targeted restoration of fast‐growing foundation species under suitable conditions.

## Introduction

Restoration of coastal foundation species has become a conservation priority because of their ecological benefits combined with their extensive global declines (Lotze et al. 2006). As human populations continue to grow in coastal areas, the impact of human activities on the foundation species that define coastal marine environments has intensified (Barbier et al. 2011, Kirwan and Megonigal 2013, Osland et al. 2019). Such impacts include effects of agriculture (Wasson et al. 2021) and urban development (Coverdale et al. 2013), diversion of freshwater inputs (Kennish 2002), and overfishing (Altieri et al. 2012) leading to trophic downgrading (Estes 2011, Kéfi 2012). Global effects of ocean acidification on coral reefs (Bellwood et al. 2004), sea level rise on tidal marshes (Thorne et al. 2018, 2018, *2018*, *2018*) and rising sea surface temperature effects on seagrass (Zimmerman and Hill 2015) and kelp forests (Muth et al. 2019, Rogers‐Bennett and Catton 2019) are some prime examples of how humans are contributing to the loss of foundation species and the services they provide. To address these various multiple stressors occurring at different spatial and temporal scales, researchers and managers are applying a diversity of restoration approaches. Intervention in the form of regulatory actions that limit harvest (Hughes et al. 2009*b*), implement water quality standards (Kennish 2002) or establish perimeters of protected habitat (e.g., Marine Protected Areas) has been effective in restoring environmental conditions conducive to the recovery of foundation species (Ling et al. 2009, Clements and Hay 2018, Geldmann et al. 2019). Transplanting/seeding foundation species (marsh plants, juvenile mangroves, oysters, corals, etc.) from areas where they are thriving to areas where they are scarce is a common restoration approach (Davis and Short 1997, Jaap 2000, Gilman and Ellison 2007, Pritchard et al. 2015, Wasson et al. 2021) used to enhance foundation species coverage and the ecosystem services and functions they provide.

Often restoration fails to bring back all the functions and services associated with foundation species, or does so slowly (Zedler and Callaway 1999, Duarte et al. 2008, Bayraktarov et al. 2016). Therefore, restoration project goals need to be developed with consideration of the life history and demography of foundation species (Montero‐Serra et al. 2018, Yando et al. 2019). For example, in regions where salt marshes (cordgrass) and mangroves co‐occur, cordgrass may be the preferred foundation species to transplant due to its fast growth, expansion, and recruitment, which expedite the restoration of ecosystem functioning (Yando et al. 2019). Generally, succession in wetland systems (e.g., salt marsh and seagrasses) is relatively rapid, making them ideal systems for understanding recovery through restoration and the ecological responses that affect a range of functions and services. Similarly, utilizing the rapid succession of fast‐growing kelps (Dayton et al. 1992, Tegner et al. 1997), active restorations in the form of artificial reefs (Reed and Schroeter 2006) and juvenile transplants (Carney et al. 2005, Layton et al. 2020) have been key in attempts to reverse widespread deforestation. In contrast, coral reef species are typically long lived and slow growing (Young and Schopmeyer 2012, Ladd et al. 2018, Ladd and Burkepile 2019); consequently, returning reefs to predisturbance conditions can take 5+ yr (Hein et al. 2021), sometimes decades (Jaap 2000, Victoria‐Salazar et al. 2017). Relatedly, certain foundation species, such as oysters, are often recruitment limited (Wasson 2016), presenting unique conservation challenges (Wasson 2020, Ridlon 2021). Thus, expectations for the rate of recovery of ecosystem services should be tailored to the life history and demography of different foundation species and habitat types.

Seagrasses are a group of marine foundation species that are in accelerated global decline (Orth 2006, Waycott 2009, Short 2011). As marine flowering plants, seagrasses are primarily limited by light availability, and most temperate seagrass species are restricted to the low intertidal or shallow subtidal zone (Zimmerman et al. 1997). Light attenuation due to poor water quality triggered by increased habitat degradation, sediment loading, eutrophication, contaminants, and other pollutants are frequently cited as the leading cause of seagrass loss (McGlathery and Sundäck 2007, van der Heide et al. 2007). To combat the widespread loss of seagrass habitat, restoration efforts are on the rise (Cunha 2012, van Katwijk 2016).

Seagrass ecosystems are highly productive and support a suite of ecosystem functions linked to valued provisioning and regulating services (Duarte 2002, Duffy 2006). For example, seagrass belowground biomass stabilizes sediment while aboveground biomass attenuates wave action (Short et al. 2011, Ondiviela et al. 2014); together these two functions provide the services of mitigating erosive forces and acting as a storm buffer. Additionally, organic particulate matter that is trapped within seagrass beds is stored in its oxygen‐depleted sediments where decomposition is relatively slow, providing the service of carbon storage. Relatedly, seagrasses both transport O_2_ to the rhizosphere, building a barrier against phytotoxins (Frederiksen and Glud 2006), and absorb large quantities of CO_2_ (Orseka et al. 2020), the latter of which has the potential to mitigate impacts of ocean acidification (Bergstrom et al. 2019, Ricart 2021). Seagrasses also provide structure that serves as nursery habitat for species of commercial importance, for example, along the U.S. West Coast, Dungeness crab (*Metacarcinus magister*) utilizes estuarine habitats, including seagrasses, during its early life stages and this particular fishery has an average annual value of over US$100 million (Hughes 2014, Grimes et al. 2020). Yet it is largely unknown whether restoration of cover by these foundation species is correlated to a related restoration of associated functions and services or how long it takes to achieve such functional recovery (but see Tay Evans and Short 2005 and Orth et al. 2020).

Given the tremendous value of seagrass ecosystems, investigations that assess both structural and functional attributes are required to evaluate whether restoration efforts can be defined as “successful.” One such study in New Hampshire’s Great Bay Estuary showed functional equivalency within 3 yr between restored and reference beds with respect to primary production, structure, and habitat use (Tay Evans and Short 2005). More recently Orth et al. (2020) used large‐scale seeding (70+ million seeds) to restore seagrass in Virginia’s recruitment‐limited coastal lagoons, where seagrass had been absent for over 70 yr. Over the past two decades, restored habitats in the coastal lagoons continued to expand and resulted in the recovery of multiple ecosystem services and even a related restoration of commercially harvested bay scallops (Orth et al. 2020). In Australia’s restored and reference seagrass (*Amphibolis antarctica*) beds, infauna abundance, belowground biomass, and sediment grain size converged within approximately 2–6 yr (Tanner et al. 2021) and in Korea (*Zostera marina*), stable isotope measures showed that food web structure between restored and reference beds were indistinguishable from one another 2 yr post‐transplanting (Park et al. 2021). Similar studies that assess both biological and biogeochemical functions are needed to gain a full understanding of ecosystem services gained from seagrass restoration.

We conducted two experimental seagrass restoration efforts and monitored success in terms of multiple structural indicators and ecosystem functions and services, in a nutrient‐loaded and highly eutrophic (Wasson 2017) estuary on the west coast of the United States from 2015 to 2018. The goals of our study were to (1) track the temporal trajectory of restored seagrass survival, expansion, and health as a critical prerequisite to recovery of ecosystem functions and (2) quantify a suite of key ecosystem functions and determine if the restored seagrass ecosystem functionality rapidly reaches the levels of naturally existing beds. First, our investigation compared restored seagrass plots vs. naturally existing reference beds for seagrass areal expansion rates and indicators of health (productivity, canopy height, above‐ and belowground biomass, shoot densities, and epiphytic algal loads). Second, we quantified ecosystem functions including biodiversity, nursery habitat, modulation of water chemistry (pH, dissolved oxygen [DO]. and water temperature), and organic carbon stocks and compared these functions individually, and collectively using a multifunctionality index (Byrnes et al. 2014) across restored, reference, and adjacent unvegetated soft‐bottom habitats. Our investigation thus serves as a model for investigating the restoration trajectory of foundation species, and the ecosystem functions they support.

## Methods

### Study site

**Fig. 1:**
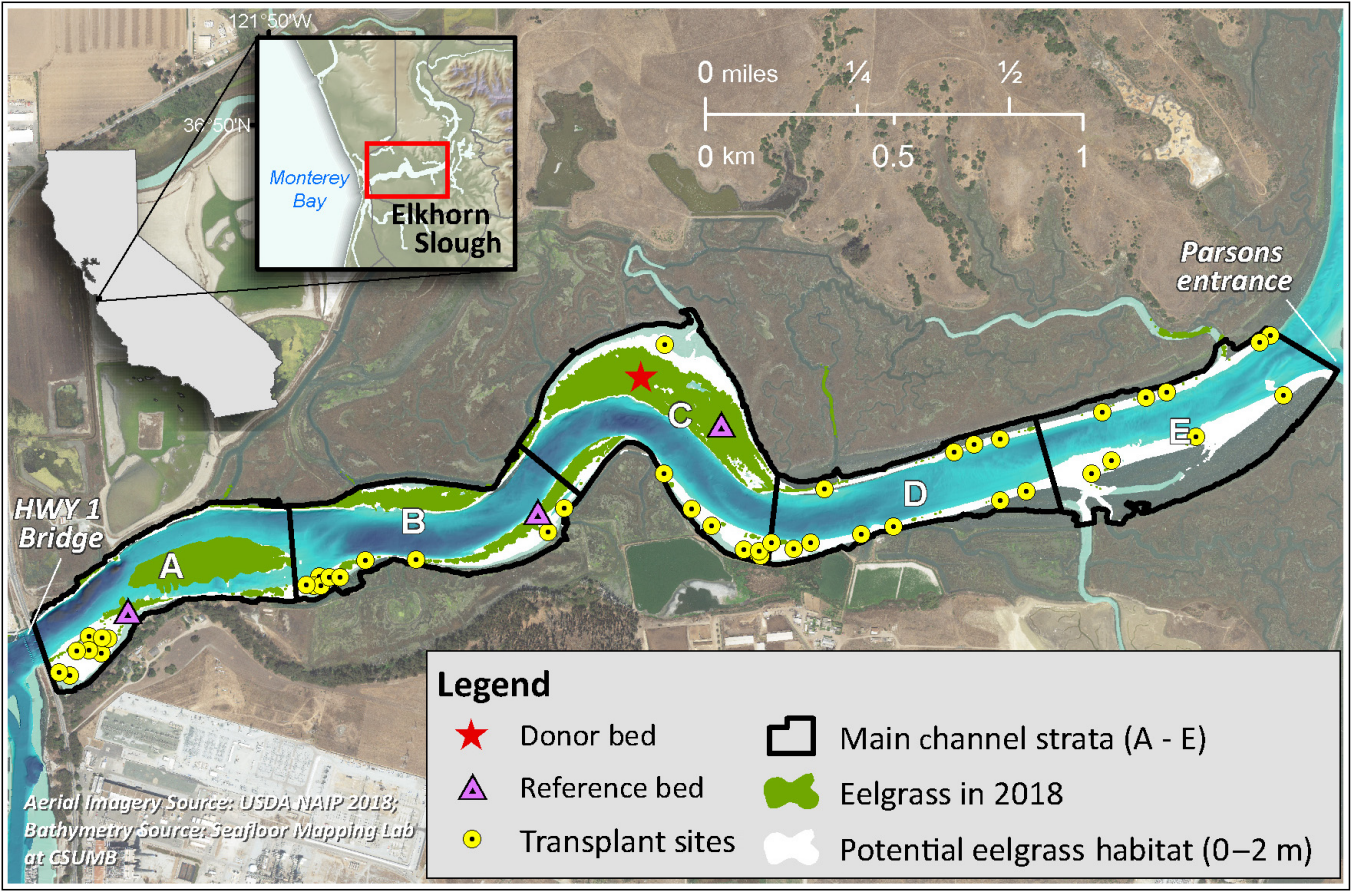
Restoration map. All sites were chosen randomly given a suite of parameters that needed to be met. All sites had to fall within a tidal elevation range that was predetermined as potential seagrass habitat (−2 to 0 m MLLW). All plots had to be >25 m from naturally occurring seagrass beds or patches. Yellow dots indicate the 2015 restoration plot locations; 2016 plots were approximately 7 m away from 2015 plots (not indicated on the map, but see Fig. S2). The donor bed was used to harvest shoots for both restorations was within Strata C (indicated with a red star). Pink triangles indicate the location of each reference meadow associated with Strata A–C.

Elkhorn Slough is an estuary located in Monterey Bay, California, USA. Highly impacted by the surrounding agricultural land, Elkhorn Slough is classified as a nutrient‐loaded system (Hughes et al. 2011, Wasson et al. 2017). Currently *Zostera marina*, commonly known as eelgrass, occurs only in the lower reaches of the estuary (Fig. 1), within the first 3 km of the main channel. This portion of the estuary is strongly marine influenced, with residence time of only about one tidal cycle and conditions that are moderately eutrophic, but less so than in the rest of the estuary (Hughes et al. 2011), yet where nutrients can approach 700 µmol (Wasson et al. 2017), ranking it as one of the most reported nutrient polluted estuaries globally (Hughes et al. 2013). In 1931, seagrass covered ~26 ha (Appendix S1: Fig. S1); thereafter, it began to decline. By the 1960s, seagrass habitat extent had plummeted to ~3 ha. Beginning in the mid‐1980s, coinciding with the local recovery of the southern sea otter (*Enhydra lutris*) population, seagrass in Elkhorn Slough began to recover to its current extent of ~15 ha. Seagrass expansion in the estuary has been associated with the recovery of sea otters that have an indirect positive effect on seagrass health through a four‐level trophic cascade (Hughes et al. 2013). Given their demonstrable impact on seagrass bed health and expansion in the system (Hughes et al. 2013), it is important to note that sea otters were highly abundant throughout our study area during the study (Tinker and Hatfield 2017, Hatfield et al. 2019).

### Small‐scale experimental restoration

We conducted two experimental seagrass restoration efforts, occurring in June 2015 and March 2016. In 2015, restoration plot locations were randomly generated using ArcGIS for Desktop v.10.2 with two qualifiers for suitable transplant sites: restoration plots had to be (1) at least 25 m from the nearest natural existing reference beds because these were areas not likely to revegetate naturally through vegetative growth or recruitment, the latter of which had been rarely observed a decade before and at the start of this study (B. Hughes, *personal observations*) and (2) within the tidal elevation range of 0 to −2 m relative to Mean Lower Low Water (MLLW), because this is the observed distribution of eelgrass in the estuary (Fig. 1). For ease of refinding and monitoring plots and to limit the number of visible PVC posts in the main channel, each 2016 restoration plot was placed approximately 7 m away from 2015 plots, still meeting the same two criteria described above (Appendix S1: Fig. S2).

To account for any physical or biological gradients within the study area we organized restoration plots to strata (A, B, C, D, and E), increasing in distance from the mouth of the estuary from strata A to E (Fig. 1). The distance from strata A to E spanned approximately 3.1 km and strata A, B, and C had nearby natural existing reference beds, hereafter referred to as reference plots, strata D and E did not and to our knowledge seagrass has never occurred in these locations. Aerial imagery and historical accounts gave us a conservative estimate for when these reference plots were established: stratum A during 1931–1947 and strata B and C during 1996–2005 (Hughes et al. 2013). For the 2015 and 2016 restoration projects, respectively, there were 8 and 12 plots in stratum A, 10 and 13 plots in stratum B, 10 and 12 plots in stratum C, 13 and 16 plots in stratum D, and 10 and 13 plots in stratum E.

All source shoots for restoration (*n* = 2,340) were harvested on SCUBA from a single donor bed, by far the largest seagrass bed in the estuary at 6.9 ha (36.816115° N, 121.766678° W). Shoots were harvested over the entire extent of this bed (~500 × 110 m) in an effort to increase genetic diversity and minimize disturbance that would otherwise result from concentrating harvest in a smaller area within the donor bed (Williams 2001, Hughes et al. 2009*a*). Rhizomes longer than 10 cm were trimmed to minimize breakage when transplanted and all shoots were trimmed to 20 cm above the most recent node to standardize starting biomass and lengths, as well as standardizing the epibiont community, essentially removing all epibionts that occur in the eelgrass canopy (Appendix S1: Fig. S3). We assured that the 20 cm above the most recent node, but above the meristem, included both sheath and blades. In 2015, 1,020 shoots were transplanted into 51, 0.25‐m^2^ plots, while in 2016, 1,320 shoots were transplanted into 66, 0.25‐m^2^ plots. Transplanted densities (20 shoots per 0.25 m^2^) were chosen based on average shoot densities of reference plots. Shoots were transplanted using a common anchoring technique: a narrow trench was built in the sediment using a hand trowel and shoots were secured in the ground with 25–30 cm galvanized garden staples (Fonseca et al. 1996a, 1996b, *1996a*, *1996b*, David and Short 1997, Orth and Harwell 1999, van Katwijk et al. 2009).

### Restoration survival and growth

To assess seagrass growth and survival, we counted the total number of vegetative shoots and recorded maximum canopy height within a quadrat placed in the initial plot area (0.25 m^2^). To assess expansion of the restored plots, we measured the maximum distance between live shoots in the plots (plot length), then took a second measurement between live shoots in a perpendicular axis (plot width) and multiplied these to obtain an estimate of plot area. Each of these parameters was monitored using SCUBA surveys of the restoration and reference plots. Monitoring for the 2015 restoration project was conducted approximately 1, 3, 6, 9, 12, 16, 24, 30, and 40 months post‐transplanting and monitoring for the 2016 restoration project was conducted approximately 1, 6, 18, and 30 months post‐transplanting. Reference plots were monitored at a minimum once each summer from 2015 to 2018. To determine whether there were differences in vegetative shoot counts between restored and reference plots we ran an ANOVA with three levels (2015 and 2016 restoration plots and reference plots) of factor “habitat type” using 2018 summer survey data.

For a more detailed assessment of seagrass growth and health, in August 2018 (~40 and 30 months post‐restoration for 2015 and 2016, respectively) we collected shoots and rhizomes (ramets) from restored and reference plots and evaluated them in the laboratory. Harvested ramets were immediately placed in fine mesh bags for transport. First, we thoroughly searched and cleared all invertebrates from harvested shoots and rhizomes for later processing (see Biodiversity of seagrass epifauna). Next, we carefully removed epiphytes from the blades and placed them in a dehydrating oven for dry mass. More details are provided in Appendix S1. For each shoot, now clean of epiphytes, we cut the rhizome at the most recent node, separating the blades from the rhizome. To compare rhizome biomass in restored and reference plots, we cut each rhizome to a standardized length of 7 cm, wrapped in a labeled foil, and placed in a dehydrating oven for 72 h to estimate dry mass (g). We cut rhizomes to 7 cm to ensure we were processing live tissue (attempting to collect more than 7 cm of rhizome in the field often results in rhizomes breaking) and because, at this length, the rhizome is approximately the same age as the shoot. Each shoot was wrapped in a labeled foil and placed in a dehydrating oven for 72 h. Dried shoots, rhizomes, and epiphytes were weighed and recorded. We analyzed data using an ANOVA with three levels (2015 and 2016 restoration plots and reference plots) of factor “habitat type” and five levels (A, B, C, D, E) of factor “strata” to compare the mean biomass of shoots, rhizomes, and epiphytes of collected ramets; a reduced model was used when the strata factor was nonsignificant at *P* > 0.05. All data were analyzed in either JMP Pro 15 (JMP, Version 15. SAS Institute Inc., Cary, North Carolina, USA, 1989–2021) or R (version 3.5.3; RStudio Team 2020) and all plots were generated using R.

### Seagrass time series

To quantify how much new seagrass habitat in Elkhorn Slough was due to restoration plot expansion vs. natural seagrass expansion, a time series was developed using geospatial data on seagrass extent. Restored seagrass area was calculated by summing the plot‐area data collected in the field from all restoration plots (2015 and 2016) in August 2018. The total extent of seagrass habitat was calculated using ArcGIS Desktop v.10.5 (Esri Inc., Redlands, California, USA) and heads‐up digitizing (approximate 1:500 scale) that incorporated aerial imagery from Google Earth Pro (*available online*)^10^ and included both restored and natural seagrass. The Google Earth imagery included a time period prior to restoration (between March 2015 and April 2016), and a time period after restoration (between February and November 2018). To get the clearest images of seagrass possible, we used multiple images from within each period that represented slightly different spatial resolutions and tidal conditions. New or expanded seagrass habitat was calculated by subtracting the pre‐restoration seagrass area from the total estimated post‐restoration area. To calculate the percent of new seagrass habitat attributed to our restoration, we used the following equation:

$$y=\left[\frac{e_{r}}{\Delta e_{t}}\right]\times 100$$

where Δ*e*_t_ is the change in total seagrass extent (restored and natural) from pre‐ to post‐restoration and *e*_r_ is the total extent restored and subsequent expansion.

### Measuring ecological functions

In order to assess the performance of various ecosystem functions across habitat types, we collected data on macrofaunal species density and abundance, seagrass epifauna community assemblage and biomass, water quality parameters (temperature, pH, and dissolved oxygen), and sediment organic carbon stocks. Because we were interested in differences across habitats, without the potential influence of spatial differences, only functional attributes within strata A, B, and C were used in the analyses since these were the strata containing both habitat types; one exception to this was our assessment of seagrass epifauna community assemblages where we sampled all restored plots (strata A–E) and compared them to reference plots in strata B–C in 2016 and strata A–C in 2018. In order to provide an assessment of the functionality of vegetated vs. unvegetated habitat, we included marked unvegetated soft‐bottom habitats, hereafter referred to as unvegetated plots, at the same depth and approximately 7 m away from each of the 2015 plots (Appendix S1: Fig. S2). Unvegetated plots were monitored concurrently with restored and reference plots.

### Biodiversity of macrofauna

To quantify biodiversity of mobile macrofauna we deployed a baited shrimp pot, hereafter referred to as a trap array, in each habitat type (unvegetated, restoration, and reference plots). More details are provided in Appendix S1. For each trapping effort, three or four trap arrays were deployed in restored (2015 and 2016), reference, and unvegetated plots in strata A, B, and C. Soak times averaged 24 h and bait was replaced every other day. We trapped a total of 21 d in summer 2016, 20 d in summer 2017, and 4 d in summer 2018. For strata A, B, and C, across all trapping years, the sample size by habitat was as follows: *n* = 26, 23, and 26 for 2015 restoration plots; *n* = 37, 30, and 29 for 2016 restoration plots; *n* = 43, 32, and 33 for reference plots; and *n* = 39, 33, and 31 for unvegetated plots. Data collection on all trapped individuals was organized by trap type (minnow trap or shrimp pot) and included species identification to the lowest taxonomic level and size. All trapped individuals were released to the location where they were initially found. When data were later analyzed, trap type was not included as a factor as data were pooled from both trap types. We assessed both species density (number of species trapped per plot) and species richness (total number of species supported per habitat). Species density, while important, does not factor in the identity of the trapped species per sampling unit (in this case, 24 h trapping efforts across 45 d). For example, a habitat that repeatedly traps species A and species B across multiple trap days would have a species density of 2, and a habitat that traps species A and species B one day and species C and D another day would also have a species density of 2 because, per effort, two species were trapped. Both overall species richness and species density provide important information about the potential for diversity to affect community structure and function. Species richness, as estimated by species accumulation curves, provides a direct assessment of the number of species that occur in a habitat, which by itself is important for conservation and management and which may also provide information about habitat complexity and the potential for functional redundancy. Species density provides important information about the interaction (particularly competition) that may regulate species in a system. In addition, differences between species density and richness will direct attention to differences in species evenness, distributions, and patterns of spatial covariance.

In order to visually compare similarities in community composition for two consecutive trapping years (summer 2016 and summer 2017), we generated a cluster diagram using a Bray‐Curtis similarity matrix using PRIMER statistical software. The end points for the Bray‐Curtis similarity matrix included habitats 2015 restoration, 2016 restoration, reference, and unvegetated plots and monitoring years 2016 and 2017. To visually assess how many species each habitat may support, we generated a species accumulation curve. To compare relative species richness across habitats, the curves were truncated to the lowest number of observations made, 33, so as to allow assessment of richness at the same level of sampling effort. We also compared species density as a function of habitat type. Species density data had many zeros and did not meet the assumptions of zero‐inflated distributions available in the generalized approach, therefore we used resampling with replacement (number of bootstrap samples = 1,000) to create distributions of means allowing comparison of habitats. Fish and invertebrate CPUE data were analyzed separately. Count data are typically non‐normal, thus to analyze catch per unit effort (CPUE), total counts per habitat per 24‐h period, we used resampling with replacement (number of bootstrap samples = 1,000) to create distributions of means allowing comparison of habitats.

Initially we tried a generalized linear model approach with either a zero‐inflated Poisson or Gamma distribution because much of our biological function data were right‐skewed, with many zeros. However, lack of model convergence pointed to the use of a more flexible and robust approach (to avoid overdispersion), therefore we used resampling with replacement (number of bootstrap samples = 1,000), hereafter referred to as “resampling,” to create distributions of means allowing comparison of habitats. A bootstrap mean of one habitat was considered significantly different from another habitat if the mean fell outside of the 95% confidence interval (CI). All data analyzed using resampling are reported below as the resampled mean ± resampled standard deviation (which is approximately equal to the standard error of the mean, SEM) followed by the 95% CI for the distribution of resampled mean, or mean_Resampled_ ± SEM (95% CI).

### Biodiversity of seagrass epifauna

To test for differences in the epifaunal community assemblage in restored (strata A–E) vs. reference plots (strata B and C), we harvested ramets from both habitats in September 2016. Collected ramets were later processed at San Francisco State University’s Estuary & Ocean Science Center in Tiburon, California. In the lab, all invertebrates were first removed from the shoots themselves using repeated freshwater dips and by hand‐picking from the mesh bag the shoots were temporarily stored in. All epifauna were identified, sorted to the lowest taxonomic level possible (usually species) and counted. These data are visualized using nonmetric multidimensional scaling (nMDS).

In August 2018, we harvested ramets to compare epifaunal counts and biomass of the species *Phyllaplysia taylori*, a marine gastropod and the marine isopod, *Pentidotea resecata*. Both are known to be important in controlling epiphytic growth on seagrass (Williams and Ruckelshaus 1993, Lewis and Boyer 2014), especially in Elkhorn Slough (Hughes et al. 2013). A single ramet from each restoration plot (strata A–E; *n* = 71) and five representative ramets from each reference plot (strata A–C; *n* = 15) were harvested and later processed at University of California, Santa Cruz’s Coastal Science Campus Laboratory. Multiple ramets had zero grazers; this was remedied in our analytical approach for both grazer counts and biomass where we used resampling with replacement (number of bootstrap samples = 1,000) to create distributions of means allowing comparison of habitats.

### Nursery function of commercially important species

We conducted additional analyses on those macrofaunal species known to use seagrass as nursery habitat and are an important commercial, subsistence, and/or recreational fishery (Hughes et al. 2014). Elkhorn Slough has been shown to be an important nursery for two rock crab species (*Cancer productus* and *M. magister*; Grimes et al. 2020) and fish groups including rockfish (*Sebastes* spp.; Hughes et al. 2014), and flatfish (*Parophrys vetulus* and *Paralichthys californicus*; Brown 2006, Hughes et al. 2015). To determine if restored seagrass had similar abundances of juveniles of these species (i.e., had similar nursery habitat characteristics), we compared the CPUE of juvenile individuals and excluded any trapped individuals larger than the maximum juvenile size class as reported in the literature (Table 1). The data were analyzed using resampling with replacement (number of bootstrap samples = 1,000) to create distributions of means allowing comparison of habitats.

**Table 1:** Total number of adult and juvenile (Juv.) individuals trapped by habitat type and data pooled across all habitat types for a subset of nursery species (Order or Family) with relatively high economic importance. The total number of both juvenile and adult individuals trapped are in bold.

Order, Family, or Species	Habitat									Juvenile size	No. individual s trapped	No. juveniles	Trapped individuals that were juveniles (%)
	REF		2015		2016		UNVEG		Total				
	Juv.	Adult	Juv.	Adult	Juv.	Adult	Juv.	Adult	By Species				
Invertebrates													
*Romaleon antennarium* (Pacific rock crab)	111	0	73	0	59	0	39	0	**282**				
*Cancer gracilis* (Slender crab)	22	5	1	1	2	0	5	0	**36**				
*Cancer productus* (Red rock crab)	70	1	17	0	14	0	17	0	**119**	<65 mm (Yamada and Groth 2016)	118	119	99%
*Metacarcinus magister* (Dungeness crab)	15	0	8	0	48	2	54	1	**128**	<50 mm (Gunderson et al. 1990)	125	128	98%
Fish													
*Sebastes* spp. (Rockfish)	11	3	11	0	19	3	2	0	**49**	<160 mm (Hughes et al. 2014)	43	49	88%
Pleuronectiformes (Flatfish)	1	0	2	0	3	0	2	0	**8**	<280 mm (Hughes et al. 2014)	8	8	100%

Notes

Habitats are REF, reference plots; 2015 restored plots; 2016 restored plots; UNVEG, unvegetated. We show the total number of individuals trapped and percentage of trapped individuals that are juveniles.

### Dynamics of key biogeochemical variables

To monitor certain water quality attributes (pH, dissolved oxygen [DO, mg/L]) and water temperature (°C), three Yellow Springs Instruments data sondes (Yellow Springs, Ohio, USA) were deployed in each habitat type within a single stratum at a time. We only had access to three sondes and therefore concentrated our sampling efforts on 2015 restored plots (to compare unvegetated and reference plots). The 2015 restored plots were chosen over 2016 plots because of their larger size. Sondes were secured to milk crates, positioned level and ~30 cm above the seafloor (Appendix S1: *Methods*). Deployment period ranged from 3 to 7 d with data collecting every 15 minutes. All sondes were calibrated using standard solutions at the Elkhorn Slough National Estuarine Research Reserve (ESNERR).

After deployment, each sonde went through a post‐calibration process to ensure that the sonde readings did not drift from the initial calibration pre‐deployment. The data were uploaded at ESNERR immediately following each deployment. Data were excluded from the analysis when the sonde was exposed at low tide and no longer submerged. Data were only used if a deployment was complete, meaning data were available for all three habitat types within strata (eight total complete deployments; two in stratum A, three in stratum B, and three in stratum C). Outliers were removed from the dataset if outside of the range of values recorded by more than two standard deviations, from a nearby long‐term water quality monitoring station during the same period using the same instrumentation (ELKVMWQ station). Analyzed data included pH data within the range of 7.5–8.5, salinity greater than 25 PSU, and dissolved oxygen 2.58–12.82 mg/L equivalent to saturation of 30–155% (96% of data fell within these ranges).

In order to compare the distributions of each water quality parameter across habitats, we used the empirical cumulative distribution function to plot the data (Wickham 2016; R package ggplot2, stat_ecdf visualization tool). To compare the distributions we conducted a nonparametric test, the Kolmogorov‐Smirnov test, to conduct a pairwise comparison of the cumulative frequency distribution (CDF) of restored and unvegetated plots against the reference plot distribution. The Kolmogorov‐Smirnov test (KS test) output includes a test statistic, *D*, the maximum difference between two cumulative distributions and a *P* value indicating the probability that the two distributions are from different populations of values. Due to the large number of samples, we were only interested in the relative difference in test statistic, *D*, between vegetated (restored and reference) and unvegetated habitats.

To quantify and compare organic carbon (OC) in the sediment of all three habitat types, approximately 20 cm of sediment was core sampled using PVC pipe (30 cm length, 5.1 cm diameter) at representative locations for each habitat. Compaction was not common and when it did occur, it was limited (<1.5 cm). We were interested in sediment carbon stock, not carbon associated with standing biomass, thus coarse living plant material (>1 cm) was manually removed. Two to three sediment cores were collected from each habitat in late 2018 (reference, *n* = 9; restoration, *n* = 8; unvegetated, *n* = 7), across strata A, B, and C. We focused our analyses on the top 10 cm of the 24 sediment cores (core depth maximum = 25 cm; mean = 10.7 cm; median = 10 cm). Once cores were extracted they were capped and taken to the laboratory where they were extruded into 2‐cm sections (intervals), wet mass determined, then dried at 60°C for 24 h. Bulk density (g/cm^3^) of each interval was calculated as the ratio of dry mass to volume of the interval (i.e., 40.54 cm^3^ for a 2‐cm interval). Each interval was rinsed of salts, dried, and cone and quartered (Lewis and McConchie 1994) and a 10 g (± 0.1000 g) subsample was randomly selected for total organic matter (TOM) analyses. TOM subsamples were acid rinsed to remove inorganic carbonates. Inorganic carbonate‐free sediments were subsampled again using cone and quartering methods (1.3 ± 0.1000 g) and placed in crucibles to burn in the muffle furnace (550°C for 3 h) for loss‐on‐ignition (LOI; Davies 1974). The difference, converted to percent loss, pre‐ and post‐combustion is the percent TOM. To convert percent TOM to percent organic carbon (OC), we used a power model ($y=0.22\chi^{1.1}$) derived using regional core data (Ward et al. 2021). Carbon storage was calculated by multiplying percent OC and the bulk density of each interval, reported as kg OC/m^3^. We used ANOVA to determine differences in organic carbon stocks across habitats. More details are provided in Appendix S1.

### Multifunctionality index

In addition to examining the above functions separately, we examined them jointly to assess multifunctionality of the habitats, which is a way to assess the synthetic qualities of the examined habitats. We used one of the most commonly used methods for quantifying multifunctionality: the averaging approach (Byrnes et al. 2014). We converted all functions from raw values with an implicit floor of the possible range in values equal to zero to standardized values with a range equal to the range in values observed. Because the ranges in values often had extreme values we truncated the range to the 95th and 5th quantiles for each function, herein this range is referred to as a function scope (Hughes et al. 2011). This approach accomplishes two goals. First, not using the scope of the data assumes values all the way to zero are possible, which is true for certain biological data but is impossible for physical factors (i.e., pH or DO). Using raw values biases the range of function values available for biological vs. physical data. Truncating the range using percentiles accomplishes the goal of diminishing the effect of extreme values in an unbiased way (same percentage of values are truncated for all functions). In order to calculate the scores for all functions where high values were considered better than low values, all measurements were subtracted from the 5th quantile value and divided by the scope (the difference between 95th and 5th quantiles), yielding values ranging from ~1 (best) to ~0 (worst). For functions where low values were considered better than high (i.e., water temperature), the measured value was subtracted from the 95th percentile and divided by the function scope, yielding values ranging from ~1 (best) to ~0 (worst). Higher relative water temperature is considered to be a negative contribution to functional performance because, while the thermal tolerance for *Z. marina* is rather wide (10–25°C; Zimmerman et al. 2015), organisms that utilize the habitat may have greater sensitivity to high temperatures. For example, when exposed to water temperatures above 22°C, the mortality rate of juvenile *M. magister* has been shown to increase (Sulkin and Mojica 1996) and a lab study found that increased temperatures (>12°C) produced the greatest deformities in *Ophiodon elongatus* hatchlings (Cook et al. 2005). Therefore, any buffering of higher relative water temperature by seagrasses is of interest because this could ameliorate conditions that would otherwise be classified as biologically stressful. Restoration plot data from the 2015 and 2016 projects were pooled into a single category: restoration plot. To look for habitat differences in multifunctionality performance, we used resampling with replacement (number of bootstrap samples = 1,000). The 95th and 5th quantiles used to standardize the data were as follows: 10.21 and 5.9 mg/L for dissolved oxygen, 8.18 and 7.78 for pH, 17.89 and 12.08°C for water temperature, and 19.34 and 4.85 kg/m^3^ for OC.

## Results

### Restoration survival and growth

**Fig. 2:**
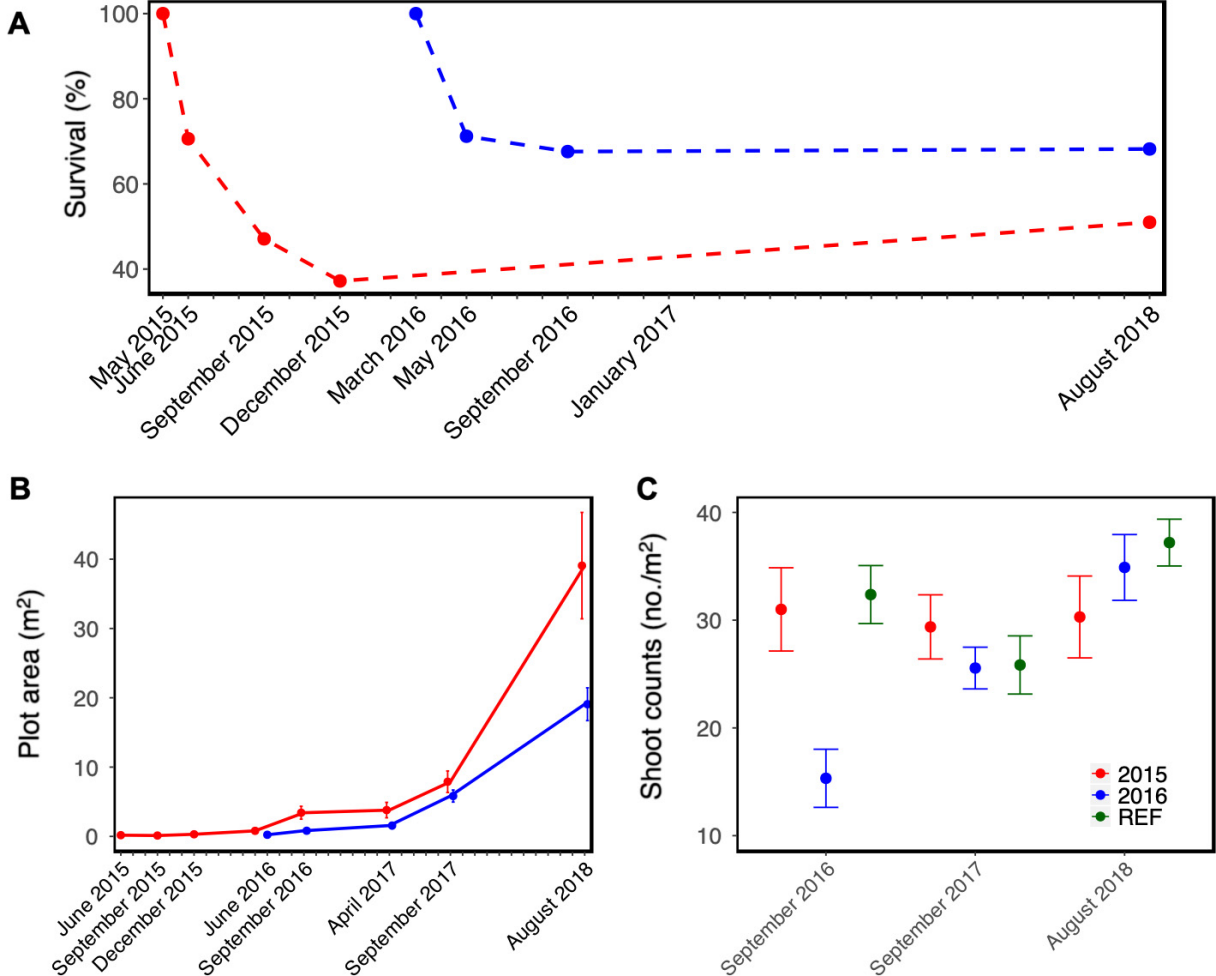
(A) Percentage of surviving restoration plots, (B) restoration plot area (mean ± SEM), and (C) shoot counts (mean ± SEM) in restored (2015 and 2016) and reference plots over time. Points show 2015 restoration plots in red, 2016 restoration plots in blue, and reference plots in green.

Our August 2018 monitoring data showed that, of 117 transplanted plots from both 2015 and 2016 restorations, 71 remained (~61%). Plot survival, measured as the percentage of plots remaining where one or more shoots were present in the initial plot area, varied by restoration effort. The 2016 restoration had lower initial (1–3 months post‐transplanting) plot mortality (32.4%, Fig. 2A) than 2015 (52.9%, Fig. 2A) and 2016 plots expanded much faster than 2015 plots (Fig. 2B). Plot mortality remained relatively unchanged for both restorations following our September surveys in 2015 and 2016 (Fig. 2A). In August 2018, we resurveyed all 2015 and 2016 restoration plots regardless of their previous status (present vs. absent) and found that seven of the 2015 plots previously considered dead/absent had seagrass within the initial transplant area, suggesting that the rhizomes may have remained intact and developed new vegetative tissue between December 2015 (when the plots were last monitored and marked as “absent”) and August 2018 (Fig. 2A). Alternatively, it is also possible that these plots were revegetated from seed potentially originating from adjacent transplanted plots with flowering shoots as negatively buoyant seeds typically do not travel far and patches of newly germinated seagrass were observed in the lower reaches of the estuary towards the end of the study period, indicating that conditions allowed for successful germination. Although, it should be noted that none of our unvegetated plots gained seagrass, which we would have expected if revegetation by seed were the mechanism.

Our restored seagrass plots expanded rapidly and contributed to seagrass expansion in this small estuary in a short period of time. One‐quarter hectare of new seagrass habitat resulted from the 2015 and 2016 restorations by year 2018. Initially, the combined area of our plots of newly transplanted seagrass totaled 12.75 m^2^ (2015) and 16.5 m^2^ (2016); by 2018 these had expanded to 1446 m^2^ (mean = 39.1 m^2^/plot) and 1068 m^2^ (mean = 19.1 m^2^/plot), respectively (Fig. 2B). Between 2014 (14.04 ha) and 2016 (14.13 ha), seagrass habitat expanded 0.09 ha in the entire estuary. Of that new habitat, ~9% (0.008 ha) can be attributed to our restoration. Between 2016 and 2018, seagrass expanded 1.49 ha, reaching 15.62 ha; our restored plots made up 17% or 0.251 ha of seagrass habitat added during this 2‐yr period.

Within 2–3 yr, in August 2018, structural attributes in restored plots were similar to reference plots. Here we report the mean and standard deviation for the measured structural attributes as well as the median to further characterize the spread of the data. Shoot counts in reference plots were generally less variable than the restoration plots (Fig. 2C), but mean shoot count in restored plots was not different from reference plots (ANOVA; *F*_2,105_ = 0.742, *P* = 0.479). Mean shoot count was 30.3 (median = 36; SD = 22.1) and 34.9 (median = 40; SD = 22.8), and mean canopy height was 155 and 138 cm for 2015 and 2016 plots, respectively (Fig. 2C, Appendix S1: Fig. S4). Mean shoot count in reference plots was 37.2 (median = 38; SD = 8.41) and mean canopy height 125 cm. There was no significant difference in mean biomass of shoots (Appendix S1: Fig. S5A; ANOVA, *F*_2,85_ = 0.186, *P* = 0.831), rhizomes (*F*_2,81_ = 0.529, *P* = 0.591), or epiphytes (*F*_2,81_ = 0.751, *P* = 0. 475) when comparing ramets harvested from reference and 2015 or 2016 restoration plots. There was a significant effect of strata on both rhizome (*F*_4,81_ = 2.989, *P* = 0.023) and epiphyte (*F*_4,81_ = 4.65, *P* < 0.0001) biomass, with a general increase in biomass moving from strata A to E (Appendix S1: Fig. S5B).

### Biodiversity of macrofauna

**Fig. 3:**
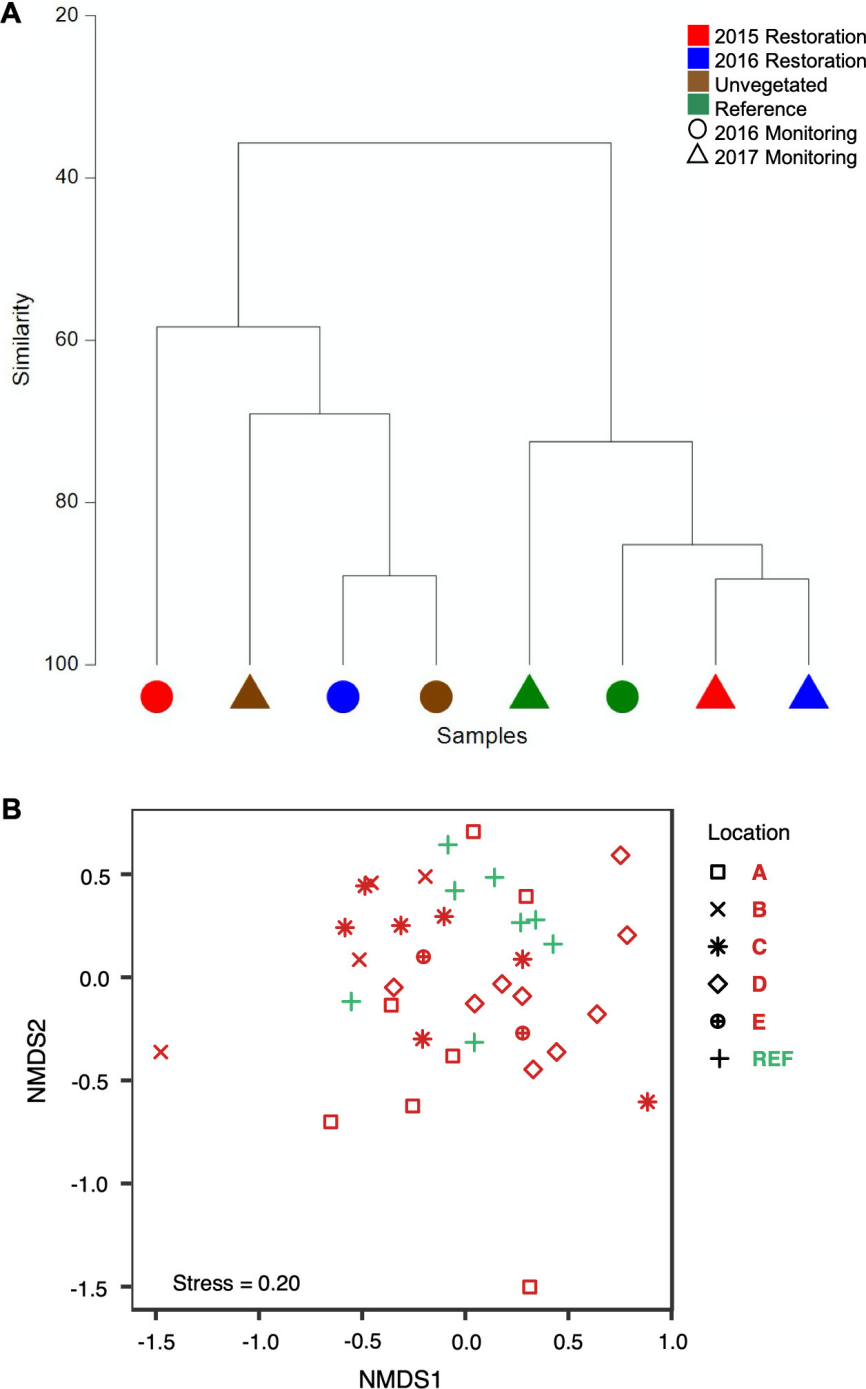
(A) Cluster diagram (Bray‐Curtis similarity matrix) for all counts of fish and invertebrate species caught in trapping years 2016 (circles) and 2017 (triangles). Communities from 2015 restoration plots are in red, 2016 restoration plots in blue, unvegetated plots in brown, and reference plots in green. (B) Nonmetric multidimensional scaling (nMDS) plot of seagrass epifauna community assemblage in restored and reference plots in the year 2016. Reference plots (strata B and C) are plotted in green as “+” and restored plots in red with each symbol representing a single stratum.

**Fig. 4:**
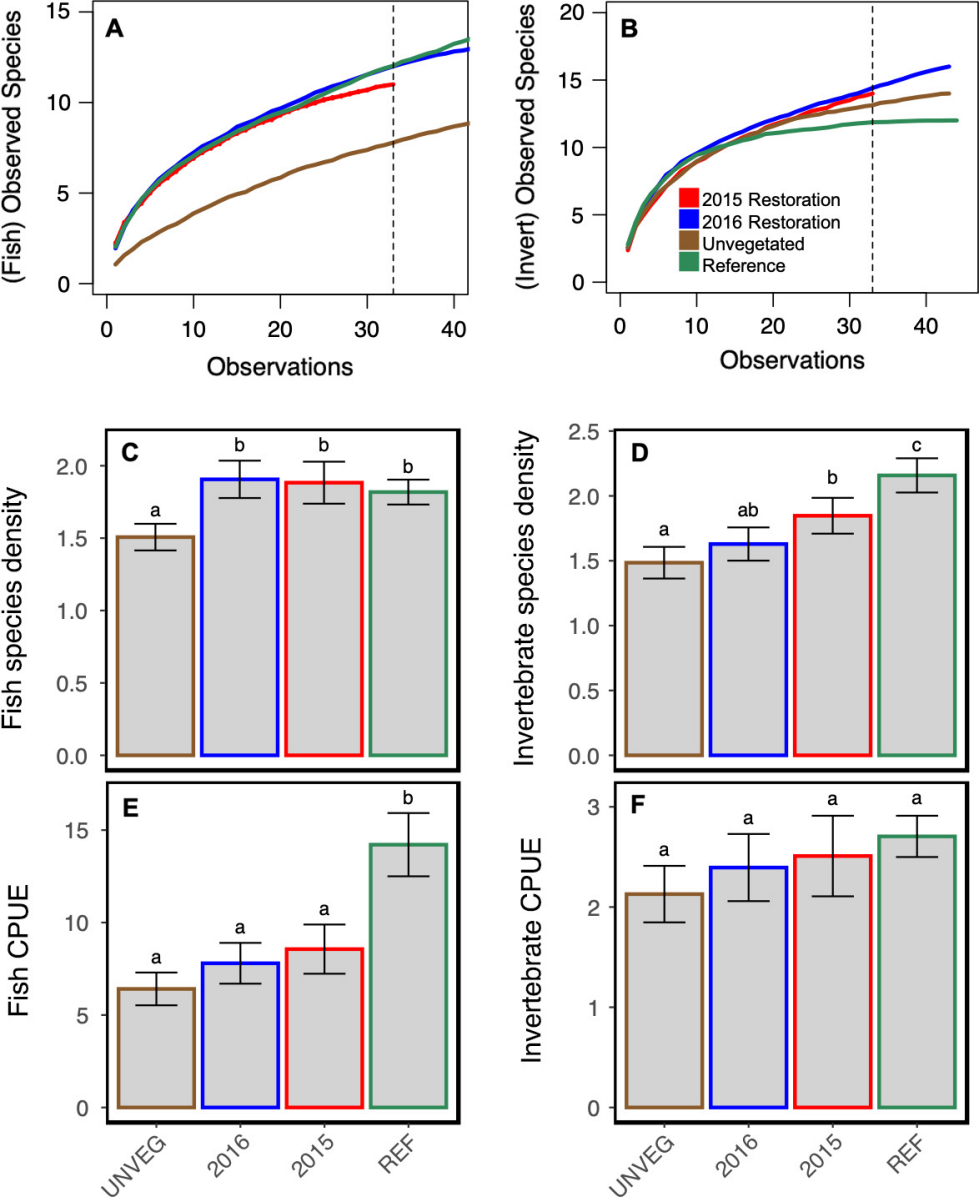
Macrofaunal diversity function. Species accumulation curves for (A) fishes and (B) invertebrates. (C) Fish and (D) invertebrate species density, quantified as the number of species trapped across 45 trapping days, plotted as the bootstrap means (±SD of the resampling distribution), which, according to the Central Limit Theorem, is approximately equal to the standard error of the raw data. The vertical dotted line represents the lowest number of observations, thus comparisons should be relative to the truncated value of 33 observations. (E) Fish and (F) invertebrate catch per unit effort (CPUE), or the mean number of individuals trapped (all species) across 45 trapping days, plotted as the bootstrap means ± SD (approximately mean ± SEM). Different letters denote significant differences (*P* ≤ 0.05) between habitats. Colors denote 2015 restoration plots in red, 2016 restoration plots in blue, unvegetated plots in brown, and reference plots in green.

Macrofaunal communities of the restored plots changed over time. The cluster diagram revealed that by summer 2017, restored plots (2015 and 2016) clustered with reference plots, whereas the year prior, the restored plots clustered with unvegetated plots (Fig. 3A). Similar community overlap was observed for seagrass epifauna (Fig. 3B). Species richness, quantified by a species accumulation curve (Appendix S1: Fig. S6) showed that at 33 observations (minimum number of observations for 2015 restoration plots), restoration plots were more species rich than reference or unvegetated plots, with unvegetated plots being the least species rich. Species accumulation curves showed close overlap for fish between vegetated habitats (restored and reference plots) and greatest differences between vegetated and unvegetated plots (Fig. 4A). In contrast, invertebrate species accumulation curves showed similarity among all habitats (Fig. 4B). These two results suggest that the overall difference between vegetated and unvegetated habitats is driven by fish (Appendix S1; Fig. S6).

Fish species density (and number of species per sample) was not different between reference (mean_Resampled_ = 1.82 ± 0.09 (1.65, 1.98)) and 2015 (mean_Resampled_ = 1.88 ± 0.14 (1.61, 2.16)) or 2016 restored plots (mean_Resampled_ = 1.91 ± 0.13 (1.65, 2.15)). However, fish species density (Fig. 4C) was higher in restored (2015 and 2016) and reference plots vs. unvegetated (mean_Resampled_ = 1.51 ± 0.09 (1.32, 1.68)). Invertebrate species density was greater in reference (mean_Resampled_ = 2.16 ± 0.136 (1.90, 2.43)) relative to restored (2015; mean_Resampled_ = 1.85 ± 0.14 (1.58, 2.12) and 2016; mean_Resampled_ = 1.63 ± 0.13 (1.37, 1.87)) and unvegetated (mean_Resampled_ = 1.48 ± 0.12 (1.24, 1.72)) plots. There was no difference in invertebrate species density between restored plots (2015 and 2016). Invertebrate species density (Fig. 4D) was greater in 2015 restoration plots relative to unvegetated plots, but no difference was detected between 2016 restoration and unvegetated plots (Fig. 4D).

Fish CPUE was greater in reference (mean_Resampled_ = 14.21 ± 1.71 (11.10, 17.69)) relative to restored (2015; mean_Resampled_ = 8.56 ± 0.13 (6.09, 11.35)) and 2016; mean_Resampled_ = 7.80 ± 1.10 (5.92, 10.22)) and unvegetated (mean_Resampled_ = 6.41 ± 0.89 (4.79, 8.17)) plots. There were no differences in fish CPUE when comparing restored (2015 and 2016) and unvegetated plots (Fig. 4E). Invertebrate CPUE did not vary as a function of habitat (Fig. 4F, Appendix S1: Table S1). A photo catalogue of the diversity of species trapped over the course of our study is presented in the Appendix S1: Fig. S7.

### Biodiversity of seagrass epifauna

The nMDS plot showed overlap in the community assemblage of seagrass epifauna between restored and reference plots. Additionally, in 2016 there was greater variation in community composition in restored plots relative to reference plots (Fig. 3B). This is largely driven by restored plots representing a broader range of environmental conditions and area relative to surveyed reference plots included in this assessment (Fig. 3B). For example, epifaunal communities in restored plots in strata further west (strata A–B) and further east (strata C–E) showed generally greater spread than strata near the surveyed reference plots (strata B and C).

In 2018, we examined a subset of species (*P. resecata* and *P. taylori*) known to be important grazers in temperate seagrass systems (Williams and Ruckelshaus 1993, Hughes et al. 2013). Results from resampling are found in Appendix S1: Fig. S8B. Counts of *P. resecata* did not vary among restored plots, but the resampled means for *P. resecata* counts in restored plots fell outside of and was greater than the 95% CI of the mean in reference plots. Counts of *P. taylori* did not vary in restored vs. reference plots. Biomass of *P. resecata* did not vary among restored plots, but the resampled means for *P. resecata* biomass in restored plots fell outside of and was greater than the 95% CI of the mean in reference plots. Biomass of *P. taylori* did not vary between restored and reference plots (see Appendix S1: Fig. S8B).

### Nursery function supports commercially important species

Generally, more individuals of commercially valuable species were trapped in reference plots compared to restored plots and in restored plots compared to unvegetated plots (Table 1). Few differences were observed between restored (2015 vs. 2016) plots (Table 1). There are notable exceptions to this; for example, 2016 restored plots and unvegetated plots trapped about three times as many *M. magister* as reference and 2015 restored plots and 2016 restored plots trapped about twice as many *Sebastes* spp. as reference and 2015 restored plots, with the fewest number trapped in unvegetated plots (*n* = 2; Table 1). Overall, the majority (79–100%) of individuals trapped across species were considered juveniles and the total number of juveniles and adults trapped by habitat are reported in Table 1.

**Fig. 5:**
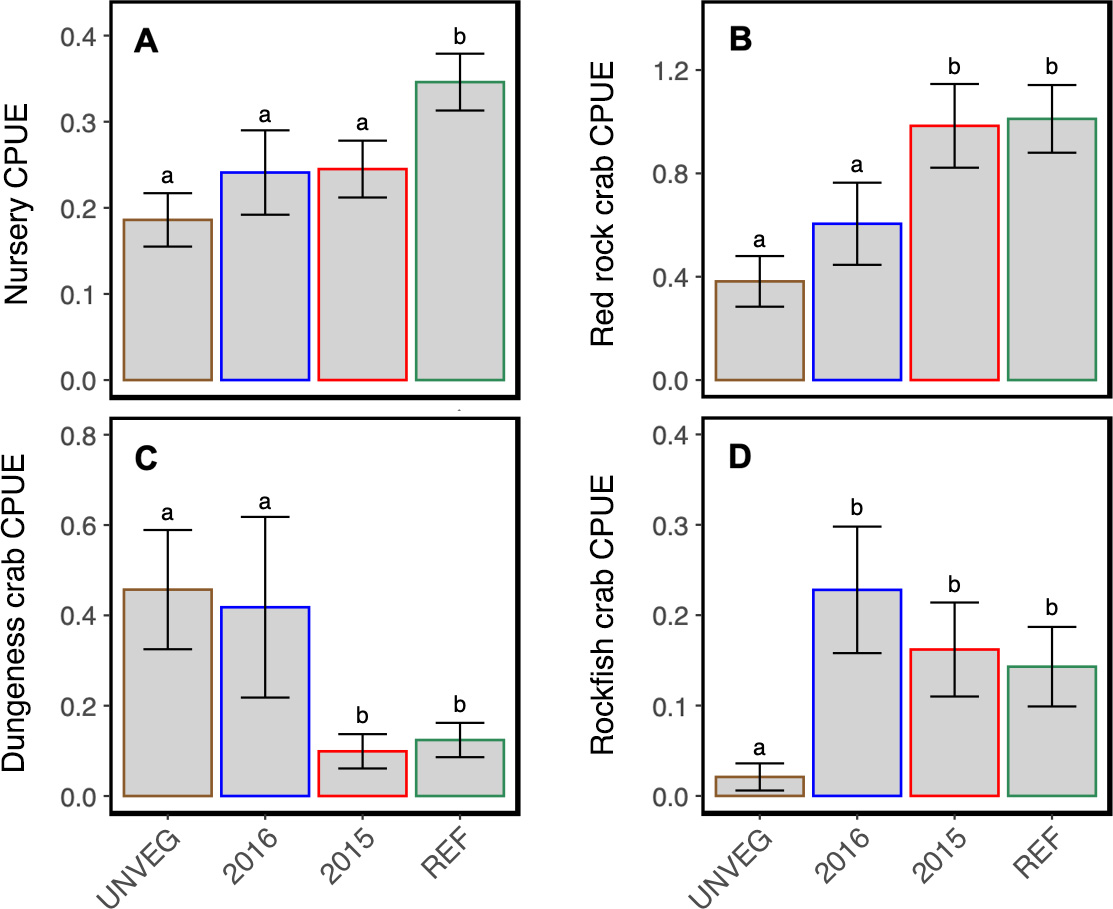
Nursery function. (A) Nursery CPUE by habitat type, quantified as the combined CPUE of all species of commercial interest (*Metacarcinus magister*, *Cancer productus*, *Sebastes* spp., *Romaleon antennarium*, *Cancer gracilis*, and Pleuronectiformes). Species‐specific CPUE by habitat type is also presented for (B) red rock crabs (*C. productus*), (C) Dungeness crabs (*M. magister*), and (D) rockfish (*Sebastes* spp.). Plotted data are the bootstrap means (of resampling distribution) ± SD (of the resampling distribution). Standard deviation of the resampling distribution is approximately equal to the standard error of the mean (SEM). Different letters denote significant differences (*P* ≤ 0.05) between habitats.

Considering the combined CPUE of all trapped species of commercial value (*M. magister*, *C. productus*, *Sebastes* spp., *R. antennarium*, *C. gracilis*, and Pleuronectiformes), we found CPUE to be greater in reference (mean_Resampled_ = 0.35 ± 0.03 (0.28, 0.41)) relative to restored (2015 (mean_Resampled_ = 0.24 ± 0.03 (0.18, 0.31)) and 2016 (mean_Resampled_ = 0.24 ± 0.05 (0.16, 0.35)) and unvegetated (mean_Resampled_ = 0.19 ± 0.03 (0.13, 0.25)) plots (Fig. 5A). No differences in nursery CPUE were detected between restored (2015 and 2016) and unvegetated plots (Fig. 5A). We found nursery function of individual commercially valuable species varied slightly by habitat, though patterns differed by species. Juvenile red rock crab (*C. productus*) CPUE was greater in reference and 2015 restoration plots relative to 2016 restoration and unvegetated plots; CPUE did not vary when comparing reference (mean_Resampled_ = 1.01 ± 0.13 (0.76, 1.27)) and 2015 restoration (mean_Resampled_ = 0.98 ± 0.16 (0.68, 1.31)) plots (Fig. 5B) or 2016 restoration (mean_Resampled_ = 0.60 ± 0.16 (0.34, 0.94)) and unvegetated (mean_Resampled_ = 0.38 ± 0.09 (0.21, 0.59)) plots (Fig. 5B). Juvenile Dungeness crab (*M. magister*) CPUE was greater in 2016 restoration and unvegetated plots relative to 2015 restoration and reference plots; CPUE did not vary when comparing 2016 restoration (mean_Resampled_ = 0.42 ± 0.20 (0.14, 0.89)) and unvegetated (mean_Resampled_ = 0.46 ± 0.13 (0.23, 0.73)) plots or 2015 restoration (mean_Resampled_ = 0.10 ± 0.04 (0.04, 0.17)) and reference (mean_Resampled_ = 0.12 ± 0.04 (0.06, 0.21)) plots (Fig. 5C). Juvenile rockfish (*Sebastes* spp.) CPUE was greater in restored (2015 and 2016) and reference plots relative to unvegetated (mean_Resampled_ = 0.02 ± 0.01 (0.0, 0.05)) plots (Fig. 5D); CPUE did not vary when comparing restored (2015; mean_Resampled_ = 0.16 ± 0.05 (0.06, 0.26) and 2016; mean_Resampled_ = 0.23 ± 0.07 (0.11, 0.37)) and reference (mean_Resampled_ = 0.14 ± 0.04 (0.07, 0.24)) plots. Juvenile flatfish were trapped in low numbers across habitats and therefore were not analyzed (Table 1).

### Dynamics of key biogeochemical variables

**Fig. 6:**
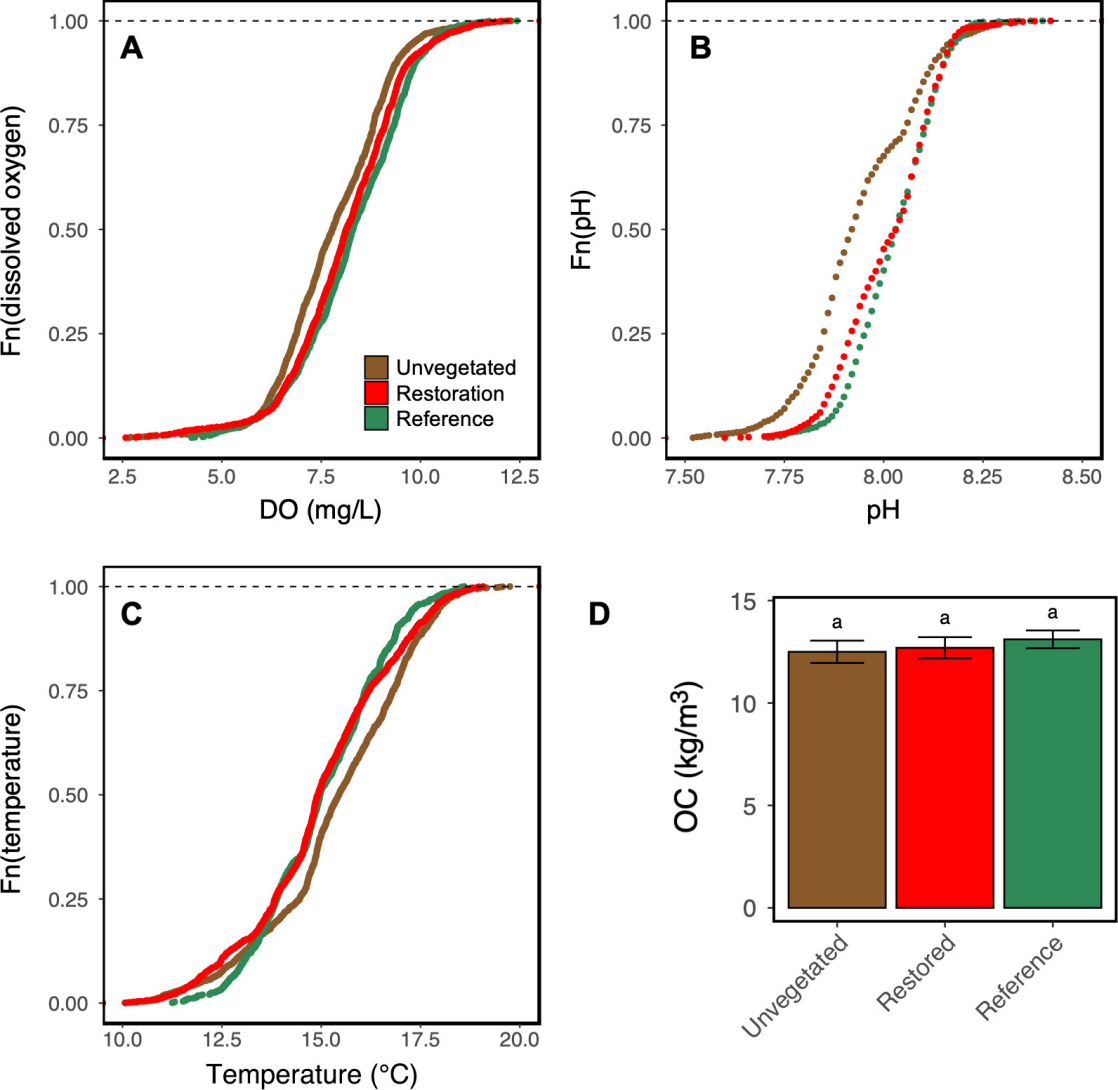
Biogeochemistry functions. Empirical cumulative distribution function curves for (A) dissolved oxygen (mg/L), (B) pH, and (C) temperature (°C). (D) Organic carbon, OC (kg/m^3^) in unvegetated and seagrass (restored and reference) plots, plotted data are OC least square means estimate ± SEM.

Generally, DO and pH were higher and water temperature lower in restored and reference plots compared to unvegetated plots. The water quality conditions observed in restored and reference plots were more similar than restored and unvegetated plots, a pattern that was clearly shown by plotting the cumulative distribution function curves and verified using Kolmogorov‐Smirnov tests (Table 2). Cumulative distribution function (CDF) curves for vegetated (restored and reference) show overlap and partial overlap, whereas there is clear separation in the CDF curves of vegetated vs. unvegetated plots (Fig. 6). The CDF curves show that DO (Fig. 6A) is generally higher in vegetated than in unvegetated plots, pH (Fig. 6B) is generally higher (less acidic) in vegetated than in unvegetated plots and water temperature (Fig. 6C) is generally lower in vegetated than unvegetated plots. The Kolmogorov–Smirnov test showed that, for each of the parameters (temperature, DO, pH), the test statistic *D* was greatest when comparing vegetated (restored and reference) to unvegetated plots and smallest when comparing restored and reference plots (Table 2).

**Table 2:** Two‐sample Kolmogorov‐Smirnov test outputs comparing water temperature, dissolved oxygen and pH between two habitats, reporting the test statistic, *D* and *P* value for each pairwise comparison.

Habitat	Water temperature (°C)		Dissolved oxygen (mg/L)		pH	
	*D*	*P*	*D*	*P*	*D*	*P*
REF × UNVEG	0.14363	7.25 × 10^−13^	0.15166	2.69 × 10^−14^	0.35117	<2.2 × 10^−16^
REF × RESTORE	0.07331	0.00189	0.07033	0.00329	0.10374	1.76 × 10^−6^
RESTORE × UNVEG	0.13417	3.56 × 10^−12^	0.13494	2.60 × 10^−12^	0.25698	<2.2 × 10^−16^

Note

Habitats are reference plots (REF), unvegetated plots (UNVEG), and restored plots (RESTORE).

Organic carbon (OC) stock did not vary as a function of habitat (Fig. 6D). Although there was no significant difference in OC across habitats, we detected significant differences in OC across strata (ANOVA; habitat, *F*_2,2_ = 0.416, *P* = 0.661; strata, *F*_2,2_ = 1658.65, *P* < 0.0001) with increases in OC with increased distance from the mouth (i.e., stratum A had the lowest OC values and stratum C the highest; see Appendix S1: Fig. S9).

### Multifunctionality index

**Fig. 7:**
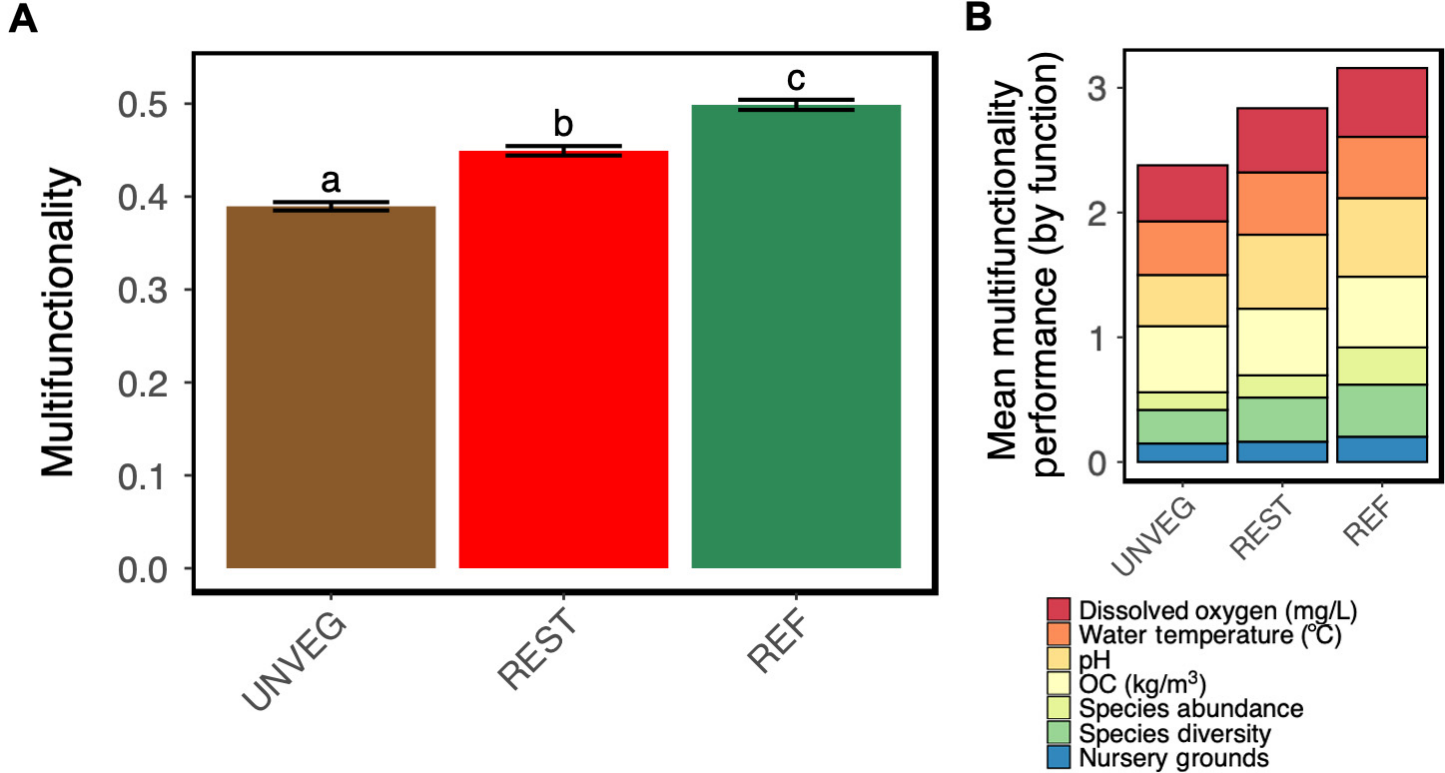
Multifunctionality. (A) Overall habitat performance plotted as the bootstrap means for each habitat (± SEM). Different letters denote significant differences between. (B) Stacked bar graph of mean multifunctionality to further describe differences in performance across habitats, color‐coded by ecosystem function to demonstrate the relative functional contribution of each of the seven functions in driving habitat differences.

Multifunctionality was significantly higher in reference plots (mean_Resampled_ = 0.498 ± 0.005 (0.49, 0.51) relative to restored plots (mean_Resampled_ = 0.45 ± 0.005 (0.44, 0.46)) and in restored plots relative to unvegetated plots (mean_Resampled_ = 0.39 ± 0.004 (0.38, 0.40) (Fig. 7A; Appendix S1: Fig. S10). The breakdown of which functions contributed substantially vs. minimally to the significant overall differences in functional performance is shown in Fig. 7B, with higher values indicating higher functional value. For example, the functional contribution of species abundance was far greater in reference plots relative to restored or unvegetated plots, whereas the functional contribution of pH was greatest in restored and reference plots and lowest in unvegetated plots, and no habitat differences were found in organic carbon storage (Appendix S1: Fig. S10).

## Discussion

### Successful seagrass restoration

Ecological restoration has been defined as “the process of assisting the recovery of an ecosystem that has been degraded, damaged, or destroyed” (SER 2004). A strategic approach to recovery is the ecological restoration of foundation species, which play a critical role in structuring communities and regulating key ecosystem processes (Ellison 2005, 2019). As a prerequisite to restoring valued functions and services, restored foundation species must survive and grow.

Seagrass restoration has proven challenging in terms of survival and growth, with a global success rate of ~37%, with success measured as an index that accounts for initial restoration survival and the longer‐term, ≥23 month, trajectory (i.e., absent, decreasing, no change, or increasing) of the restoration (van Katwijk et al. 2016). Our small‐scale restoration project had a success rate, calculated as the percentage of plots remaining 30–40 months post‐transplanting, of 61%, far exceeding the global average for both small (22%) and large (42%) scale seagrass restorations (van Katwijk et al. 2016) and we observed rapid expansion of our restored plots in total area (~8,500%). Overall transplant survivorship and spread was remarkably high in our study, indicating that our restoration efforts were successful and on par with the well‐documented outcomes of larger scale restorations (Tay Evans and Short 2005, Leschen and Ford 2010, McGlathery et al. 2012). We attribute our high success to a selection of factors linked to site and design, which compliments the findings of others that emphasize the need to address adverse conditions first or fully assess site suitability prior to transplanting or seeding to improve the likelihood of restoration success (Fonseca and Kenworthy 1982, van Katwijk and Hermus 2000, Bell et al. 2008, Thom et al. 2012, 2018*a*, *b*).

Our success in Elkhorn Slough is likely due to many factors related to the site itself and our restoration design. Despite high nitrate concentrations and the proliferation of macroalgal blooms (Wasson et al. 2017), the natural recovery of seagrass in Elkhorn Slough over the past 30 years (Appendix S1: Fig. S1) is evidence of an overall improvement of environmental conditions conducive to seagrass growth and expansion. Elkhorn Slough is also the only estuary along the California coast with a large (100+) population of resident sea otters, *Enhydra lutris* (Tinker and Hatfield 2017). Sea otters have been shown to have strong top‐down effects on the health of seagrass beds in Elkhorn Slough (Hughes et al. 2013). The return of this keystone predator to the system has been linked to natural seagrass recovery and may have contributed indirectly to our positive restoration outcomes by improving seagrass health and promoting natural expansion throughout the system. Additionally, we transplanted within a narrow depth range of 0 to −2 m MLLW. Within this tidal range, restoration plots were exposed infrequently at low tides and had sufficient light availability at high tide, nearly eliminating plot mortality linked to desiccation stress or high light attenuation. Lastly, our transplanting method of anchoring ramets (shoots with intact rhizomes) to the substrate has been shown to have higher rates of success than other transplanting methods (Park and Lee 2007, Bell et al. 2008, Eriander et al. 2016, van Katwijk et al. 2016). All of these factors associated with site suitability and restoration design likely contributed greatly to our restoration success. By contrast, attempts to restore seagrass in other degraded systems in the region have had minimal success. For example, in Morro Bay, California. 95% of eelgrass has been lost and efforts to restore have had variable success; this may be linked to the degraded state of natural meadows (Harenčár et al. 2018), massive system‐wide erosion and sediment resuspension shifting tidal elevations throughout the estuary (Walter et al. 2020), or restoration design.

### Restoring multiple functions of foundation species

Foundation species provide ecosystem structure (Dayton 1972) and structure begets ecosystem function (Dobson and Bradshaw 1997, Bruno and Stachowicz 2003). For example, successful large‐scale seeding efforts in Chesapeake Bay led to the restoration of seagrass habitat and associated functions and services (i.e., decreasing turbidity levels, increasing carbon stocks, habitat provisioning; Orth et al. 2020). Therefore, ecological restoration of declining foundation species, such as seagrasses (Short et al. 2011), not only supports recovery of the vegetation itself, but potentially has the added benefit of enhancing biodiversity and ecosystem functions (Tay Evans and Short 2005, Benayas et al. 2009, Angelini et al. 2015). The primary focus of seagrass restoration monitoring has been the foundation species itself (structural attributes) and to determine how site characteristics (van Katwijk et al. 2009, Thom et al. 2018, 2018, *2018*, *2018*) and methodology (transplantation or seeding techniques; Park and Lee 2007, Bell et al. 2008, Eriander et al. 2016) affect survival. In select restoration studies, structural attributes and a few additional associated ecosystem functions (canopy friction and sediment movement [Fonseca and Fisher 1986], faunal communities [Fonseca et al. 1996a, 1996b, *1996a*, *1996b*, Leschen et al. 2010], carbon and nitrogen sequestration [McGlathery et al. 2012, Greiner et al. 2013, Reynolds et al. 2016]) have been assessed. Yet, few studies have simultaneously tracked multiple structural and functional attributes of biological and biogeochemical importance. Our study is the first comprehensive investigation of eelgrass restoration on the eastern Pacific Coast to track structural attributes and such a large suite of biological and biogeochemical ecosystem functions permitting a comprehensive assessment of functionality. This approach of fully characterizing the success of our ecological restoration was powerful and if sufficient funding is available for monitoring, could be broadly applied to restorations of other foundation species. To infer functional recovery in other systems, we recommend similar focal studies be conducted to validate that certain functions are enhanced with restorations elsewhere. Here, we review the highlights of the multiple functions we evaluated.

Generally, biological functions recovered rapidly. This is likely due to our restoration method, which immediately added habitat structure to support species use, and the rapid growth and expansion of restored plots, which quickly resembled reference plots (i.e., shoot densities, canopy height, biomass). For example, we observed rapid colonization critical mesograzers in 2016 (Healey and Hovel 2004, Hughes et al. 2013, Lefcheck and Marion 2017) and greater species richness in restored habitats due in large part to the greater geographic area sampled in restored (strata A–E) vs. reference (strata B–C) habitats. Interestingly, by 2018, mesograzers *P. resecata* and *P. taylori* were more abundant in restored plots and *P. resecata* biomass was greater in restored vs. reference plots. This could be due to relatively fewer known predators (i.e., Cancridae crabs) of mesograzers found in restored plots compared to reference plots. Additionally, within 2–3 yr, restored plot nursery function, macrofaunal community composition, fish and invertebrate species density, and fish abundance, recovered to levels at or nearing those observed in reference plots.

Our expectation that nursery function would be greatest for reference plots was met and this was largely driven by many more predatory crabs in reference vs. restored plots (Table 1). Habitat differences in nursery function likely emerged due to a preference by juveniles for structured habitat (i.e., red rock crab) and/or refugia (i.e., rockfish; Dungeness crab). Red rock crab CPUE increased with habitat structure with the highest CPUE values observed in 2015 restoration and reference plots and the lowest values in unvegetated and 2016 restoration plots: we expect CPUE in 2016 plots to increase as the plots continue to expand. Similarly, juvenile rockfish are known to prefer structured habitat (Love and Carr 1991, Olson et al. 2019) and our data support these findings as rockfish were found more commonly in vegetated (restored and reference) vs. unvegetated habitat. Alternatively, Dungeness crab appear to prefer unvegetated habitat or smaller patches of seagrass (2016 restoration plots) over larger patches (2015 restoration plots) or continuous beds (reference plots). Preference for unstructured habitat by Dungeness crab is well documented (Holsman and McDonald 2006, Holsman et al. 2010) and in Elkhorn Slough, sea otters, a common predator of Dungeness in the system, have depressed crab size and abundance in unvegetated habitats (Grimes et al. 2020). Therefore, we suspect that Dungeness crabs utilize the smaller 2016 restoration plots as temporary refuge from predators.

As restored plots matured and expanded, macrofaunal diversity and community composition progressed towards reference plot levels. Restored and reference plots supported higher fish species densities than unvegetated plots, complimenting previous work (Ruesink et al. 2019), but fish CPUE was lower in restored plots and may be related to the size or “patchiness” of restored plots compared to the continuous reference beds. Invertebrate species richness increased with habitat structure and maturity (with unvegetated as structureless and restored as structured but less mature than reference), whereas invertebrate CPUE did not vary by habitat, suggestive that species evenness is lowest in highly structured habitats (i.e., reference). Generally, macrofaunal diversity in restored plots is moving towards levels observed in reference plots, this is further supported by the cluster diagram (Fig. 3A), showing that similarity in macrofaunal community composition of restored plots shifted from unvegetated to reference plots over time. In summary, certain biological functions in restored plots are currently performing at or near levels observed in reference plots while others are higher than unvegetated plots and lower than reference plots. We expect such functions to edge towards reference plots as restored plots continue to expand.

While biological functions were fast to emerge, biogeochemical functions either did not vary across habitats (i.e., organic carbon stocks, OC) or were more subtle and nuanced (i.e., DO, pH, water temperature). Because water chemistry is highly context dependent, an assortment of factors (incoming vs. outgoing tides, distance from mouth, amount of water in the basin, tidal height, nutrients) could mask the signal of eelgrass modulating water chemistry. Due to the wealth of data available in this study, we were able to use CDF curves to better visualize differences in pH, DO, and water temperature across restored, reference, and unvegetated habitats. By plotting data from all eight deployments, we captured a range of hydrodynamic conditions and still managed to detect differences between unvegetated and vegetated habitats. This was further supported by the K‐S test, which showed restored and reference plots were statistically more similar to one another than either were to unvegetated plots. Generally, water temperature was lower and pH and DO higher in restored and reference plots compared to unvegetated plots. Last, we expected restored and reference plots to have greater OC than unvegetated plots, and instead observed no detectable differences among habitats but a near doubling of OC with increased distance from mouth. This is likely due to a change in grain size moving upstream, from predominantly sandy to silty (Ward et al. 2021), and shows that in Elkhorn Slough, the ability of eelgrass to shift sediment properties is context dependent (Appendix S1: Fig. S9).

Our use of sophisticated visualizations and analyses allowed us to better evaluate habitat differences within (e.g., CDF curves, species accumulation curves) and across functions (multifunctionality index). For example, by plotting both species density and species accumulation curves, we were able to assess the expected number of species per plot type and the number of species supported per habitat, respectively. Discrepancies between species density and species accumulation curves can be due to differences in overall abundances or evenness and are informative in assessing biodiversity. As stated above, CDF curves allowed us to compare the distribution of water quality parameters between habitats in a holistic way that is biologically relevant; for example, mean pH across habitats may not be different, but if the frequency of observations for pH in vegetated habitats is higher than in unvegetated habitats, these detailed patterns may have ecological significance. Finally, the multifunctionality index was useful in visualizing our overall finding that the cumulative functional performance of restored habitats was intermediate: higher than unvegetated and lower than reference. The index also allowed us to determine, in a standardized way, the relative contributions of each function in driving differences between habitats (mainly species diversity, pH, and DO).

The rapidity with which functioning was enhanced in Elkhorn Slough illustrates the potential for successful ecological restoration of a foundation species. Fast‐growing foundation species such as seagrasses are able to restore ecosystem function faster than foundation species that take years to reach maturity, or than species for which old tissue plays a large role in engineering effects (Montero‐Serra et al. 2018). For example, in semideciduous riparian tropical forests, restored habitats can take up to 70 yr to reach old growth forest levels of species richness (Suganuma and Durigan 2015). Similarly, in coral reef systems, coral transplants assist in the recovery of rugose structures and yet functional recovery is slow to follow due to slow growth of such reef‐building coral species (Ladd et al. 2019). In addition to contrasts among species with different life histories, there are contrasts among functions; some, such as providing structured habitat for animals, may be achieved more rapidly than others, such as carbon storage. As more restoration projects include monitoring of multiple ecosystem functions as we have done here, conservation planners can form realistic expectations of the rate of recovery of different important ecosystem services across contrasting foundation species.

## Supporting information

Appendix S1

## Data Availability

Data (Beheshti 2021) are available in the Dryad Digital Repository: https://doi.org/10.7291/D1M96K

## Footnotes

^10^

https://www.google.com/earth/versions/
