# Supplementary material for: Rapid enhancement of multiple ecosystem services following the restoration of a coastal foundation species
Source: Ecol Appl. 2021 Oct 25;32(1):e02466. doi: 10.1002/eap.2466 (PMC9285811; doi:10.1002/eap.2466)
Supplement: Supplementary file 1 — Appendix S1 [file EAP-32-0-s001.pdf]

Supporting Information. Beheshti, K.M., S.L. Williams, K.E. Boyer, C. Endris, A. Clemons, T. Grimes, K. Wasson, and B.B. Hughes. 2021. Rapid enhancement of multiple ecosystem services following the restoration of a coastal foundation species. Ecological Applications.

Appendix S1: Technical details and supplemental figures and tables

Species Density

Fish or Invert	Habitat	Resampled Mean	Std. Dev. (~SEM)	95% CI	Habitat Diff.
Fish	UNVEG	1.51	0.09	(1.32, 1.68)	a
Fish	2016	1.91	0.13	(1.65, 2.15)	b
Fish	2015	1.88	0.15	(1.61, 2.16)	b
Fish	REF	1.82	0.09	(1.65, 1.98)	b
Invert	UNVEG	1.49	0.12	(1.24, 1.72)	a
Invert	2016	1.63	0.13	(1.37, 1.87)	ab
Invert	2015	1.85	0.14	(1.58, 2.12)	bc
Invert	REF	2.16	0.13	(1.90, 2.43)	c

CPUE

Fish or Invert	Habitat	Resampled Mean	Std. Dev. (~SEM)	95% CI	Habitat Diff.
Fish	UNVEG	6.41	0.89	(4.79, 8.17)	a
Fish	2016	7.80	1.10	(5.92, 10.22)	a
Fish	2015	8.56	1.33	(6.09, 11.35)	a
Fish	REF	14.21	1.71	(11.10, 17.69)	b
Invert	UNVEG	2.13	0.28	(1.58, 2.73)	a
Invert	2016	2.39	0.34	(1.78, 3.10)	a
Invert	2015	2.51	0.40	(1.85, 3.45)	a
Invert	REF	2.71	0.21	(2.30, 3.09)	a

Table S1. Macrofaunal Diversity (richness and abundance). (Top) Species density for fish and invertebrates and (Bottom) fish and invertebrate CPUE. Reported data include the resampled mean and standard deviation (approximately equal to standard error), the 95% CI, and significant differences ($p<0.05$) between habitats, denoted by different letters. Each of the four habitat types are shown in the order that they are plotted in the main text ("UNVEG"=Unvegetated, "2016"=2016 Restoration, "2015"=2015 Restoration, and "REF"=Reference).

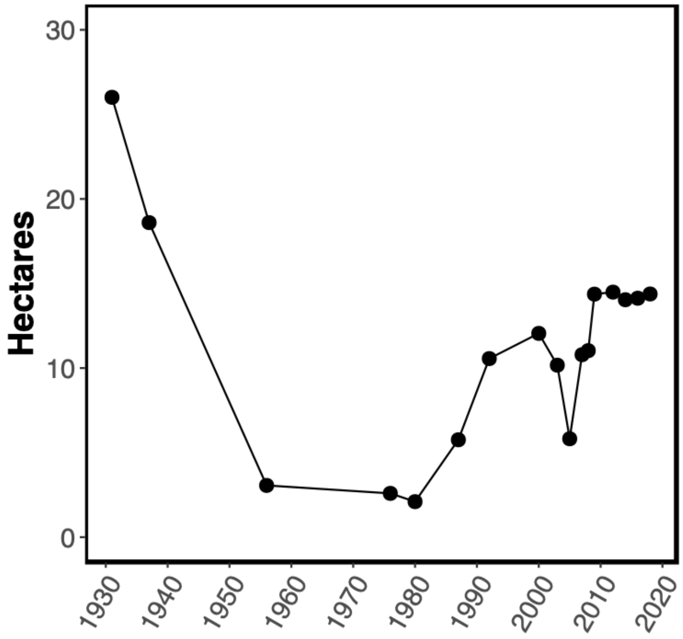

Figure S1. Seagrass Time Series from 1931 to 2018 showing seagrass aerial extent in Elkhorn Slough, CA starting at 26.01 hectares in 1931 to 15.62 in 2018. By the 1960s seagrass extent dropped as low as ~3 hectares. These data are based on aerial imagery data. See Appendix S1: Methods for details.

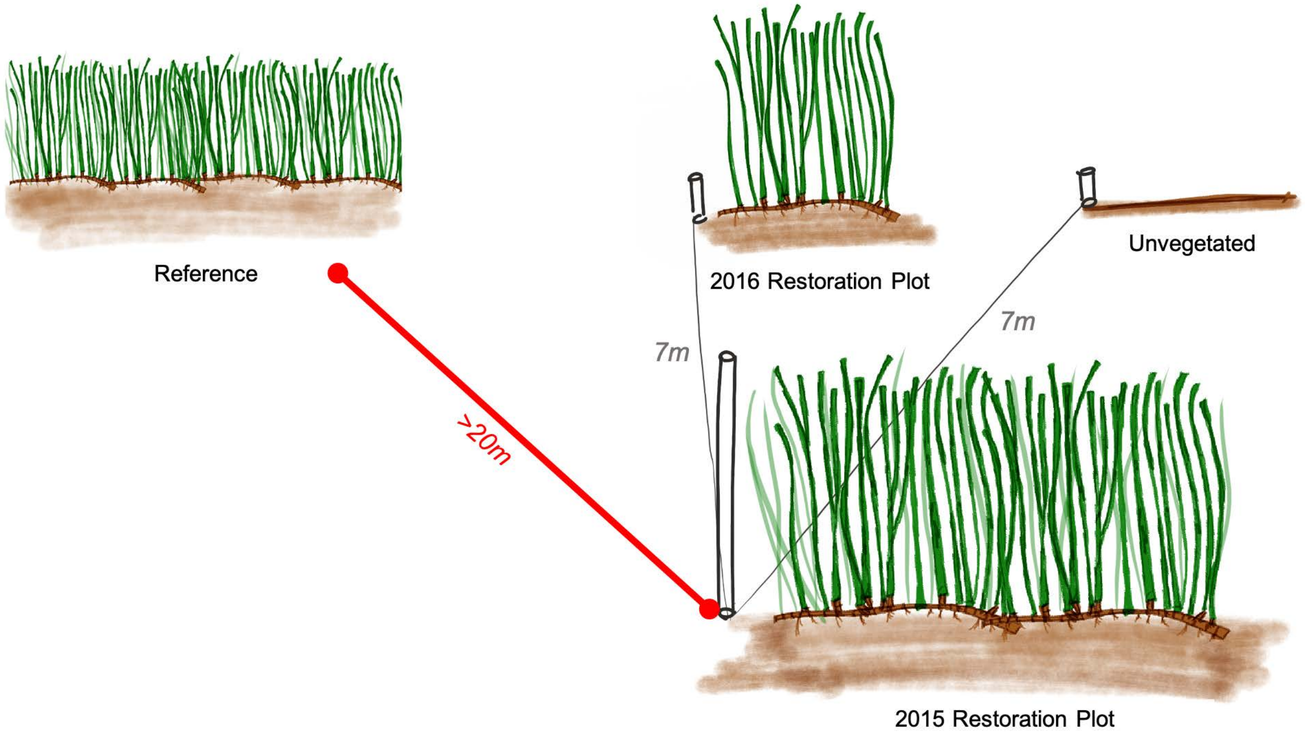

Figure S2. Schematic of restoration design (2015 and 2016). Restoration plots were ~7 m away and reference beds were at least 25 m from restored plots at the time of transplanting. Illustration by Kathryn Beheshti.

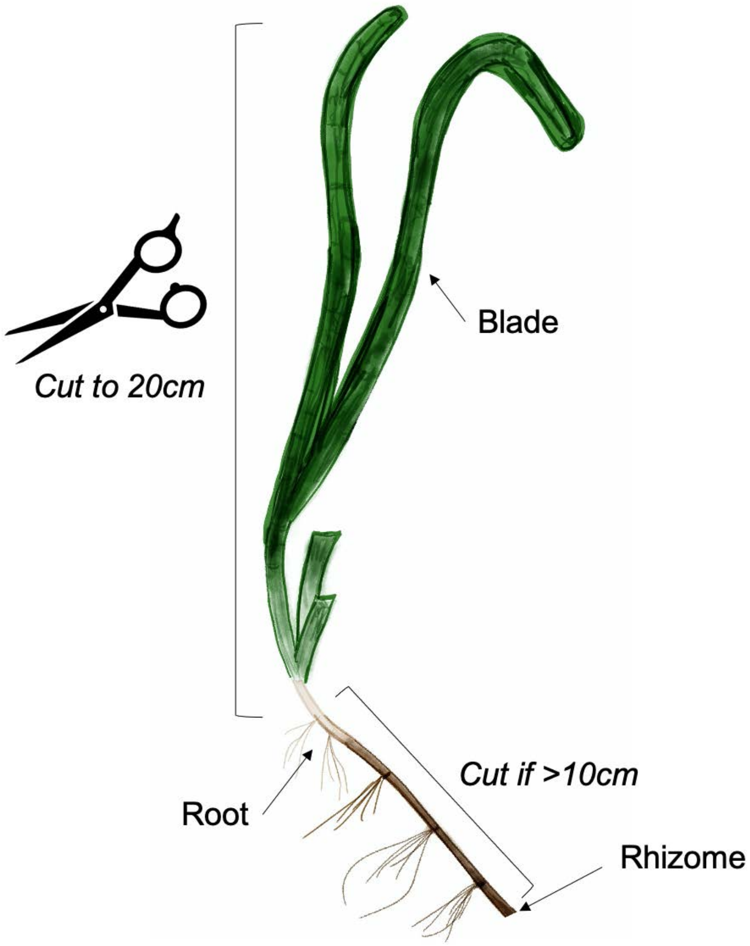

Figure S3. *Zostera marina* morphology and showing where shoots and rhizomes were trimmed for transplanting (shoot, 20 cm; rhizome, 10 cm) and later lab processing (rhizome, 7 cm). Illustration by Kathryn Beheshti.

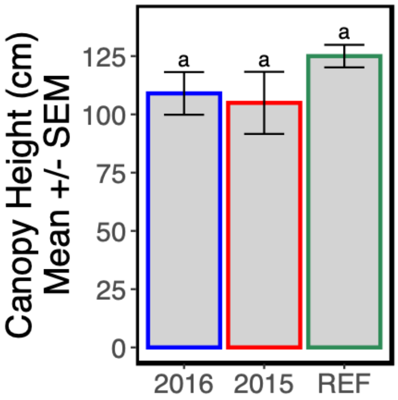

Figure S4. Expansion of reported structural attributes of restored (2015 and 2016) and reference plots from August 2018 monitoring. Canopy height (cm) (per 0.25 cm2) for August 2018 monitoring effort for 2015 and 2016 restoration plots and reference bed plots. Presented canopy height data is based on raw data. Different letters denote significant differences ($p<0.05$) between habitats.

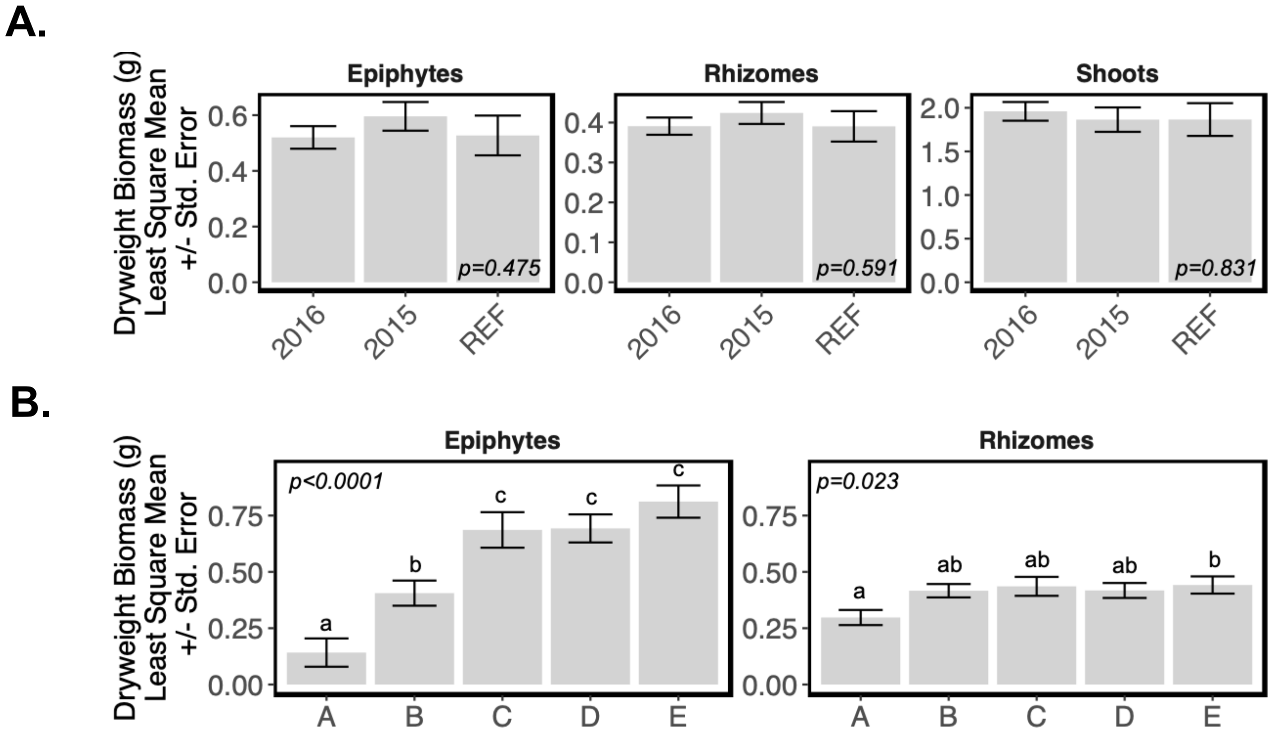

Figure S5. Shoot and rhizome mean biomass and epiphytic algae/diatom load by A) habitat type and B) strata. Plotted is the biomass data (least square mean estimate ± standard error) from August 2018, 2 and 3 years post-transplantation for the 2015 and 2016 restorations. ANOVA p-values are reported in the lower right (A) and upper left (B) hand corners. See Appendix S1: Methods for details.

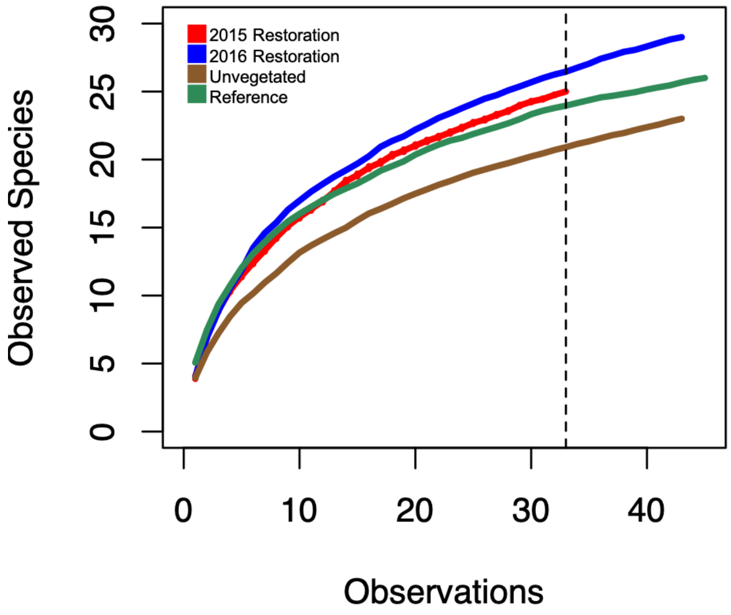

Figure S6. Species accumulation curve for all trapped species across all trapping years (2016, 2017, and 2018). The vertical dotted line represents where the lowest number of observations were, and therefore the comparison should be relative to the truncated value of 33 observations.

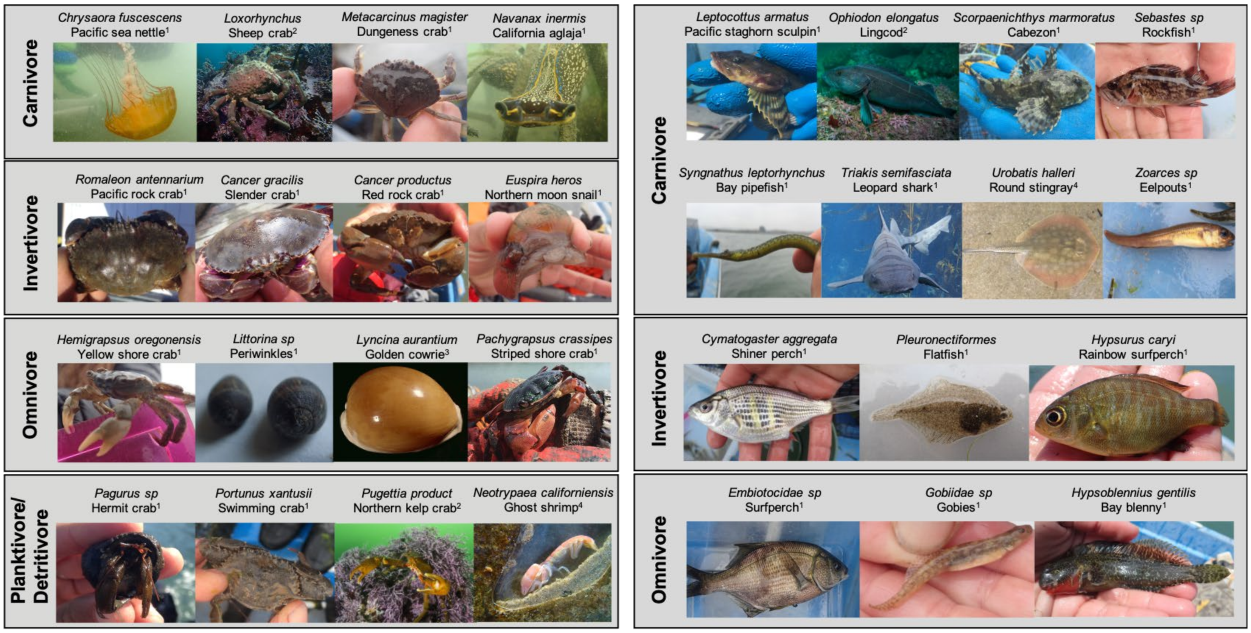

Figure S7. Most commonly trapped species of invertebrates (left) and fishes (right) organized by functional group. Photo credit: (1) Kat Beheshti, (2) Michael Langhans, (3) National Geographic, (4) Monterey Bay Aquarium.

A.

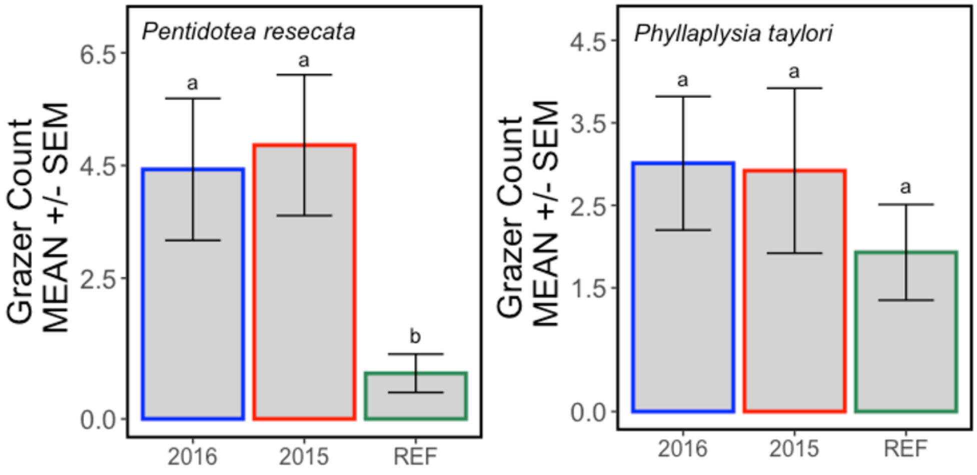

Grazer Count

Species	Habitat	Resampled Mean	Std. Dev. (~SEM)	95% CI	Habitat Diff.
Pentidotea resecata	2016	4.43	1.26	(2.13, 7.21)	a
Pentidotea resecata	2015	4.86	1.25	(2.50, 7.32)	a
Pentidotea resecata	REF	0.81	0.34	(0.20, 1.50)	b
Phyllaplysia taylori	2016	3.01	0.81	(1.53, 4.74)	a
Phyllaplysia taylori	2015	2.92	1.00	(1.09, 5.23)	a
Phyllaplysia taylori	REF	1.93	0.58	(1.00, 3.10)	a

B.

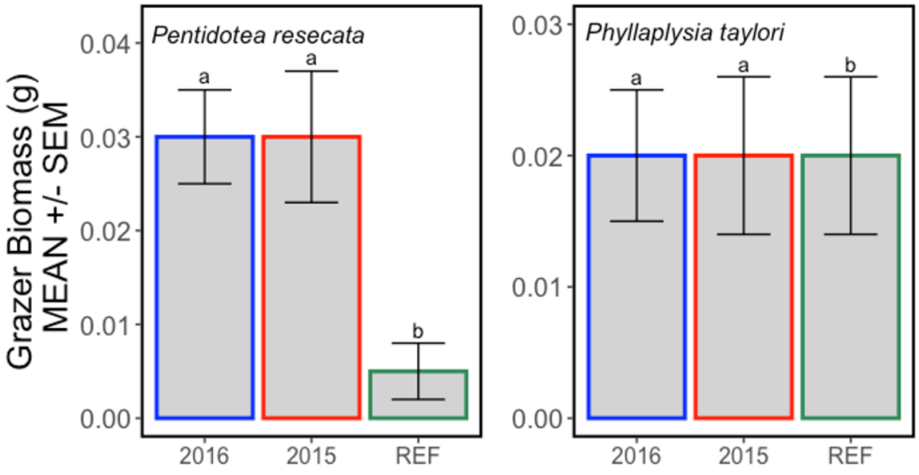

Grazer Biomass

Species	Habitat	Resampled Mean	Std. Dev. (~SEM)	95% CI	Habitat Diff.
Pentidotea resecata	2016	0.03	0.005	(0.02, 0.04)	a
Pentidotea resecata	2015	0.03	0.007	(0.01, 0.04)	a
Pentidotea resecata	REF	0.005	0.003	(0.0004, 0.01)	b
Phyllaplysia taylori	2016	0.02	0.005	(0.01, 0.03)	a
Phyllaplysia taylori	2015	0.02	0.006	(0.007, 0.03)	a
Phyllaplysia taylori	REF	0.02	0.006	(0.01, 0.04)	a

Figure S8. Resampled means for A) counts and B) biomass (g, dry weight) of epifaunal grazers *Pentidotea resecata* and *Phyllaplysia taylori* by habitat type. The data presented is from the August 2018 monitoring effort. Different letters signify significant differences between habitat types ("2015" = 2015 restoration, "2016" = 2016 restoration and "REF" = reference plots). Count data are plotted as the bootstrap means (of resampling distribution) ± standard deviation (of the resampling distribution), which is approximately equal to SEM. We have also included tables associated with each of the graphs that show the Mean, Std Dev (~SEM), 95% CI, and significant habitat differences ($p<0.05$).

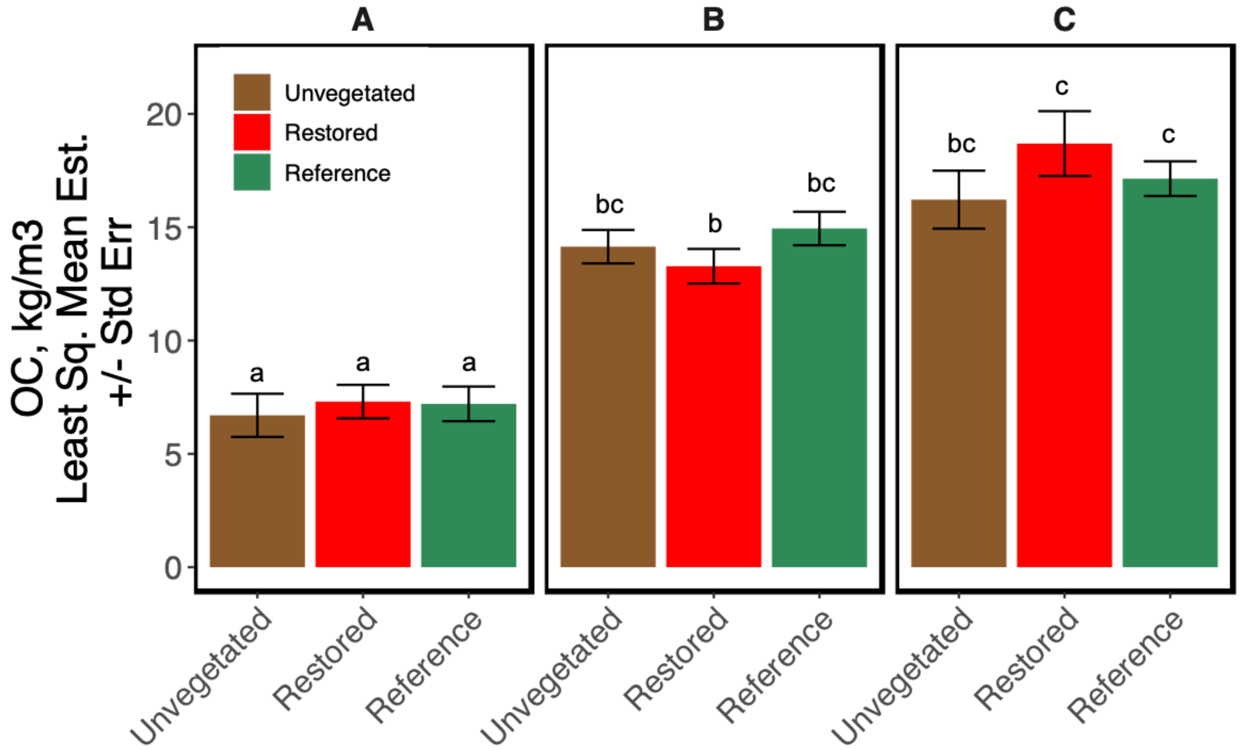

Figure S9. Organic carbon stocks (OC, kg/m^3) by habitat and strata, plotted as the Least Square Means Estimate ± SEM. Different letters denote significant differences between habitats according to Tukey's HSD post-hoc tests. Unvegetated plots are in brown, restored plots in red, and reference plots in green. Stratum A (nearest to mouth of estuary) is characteristically sandy while stratum C is silty.

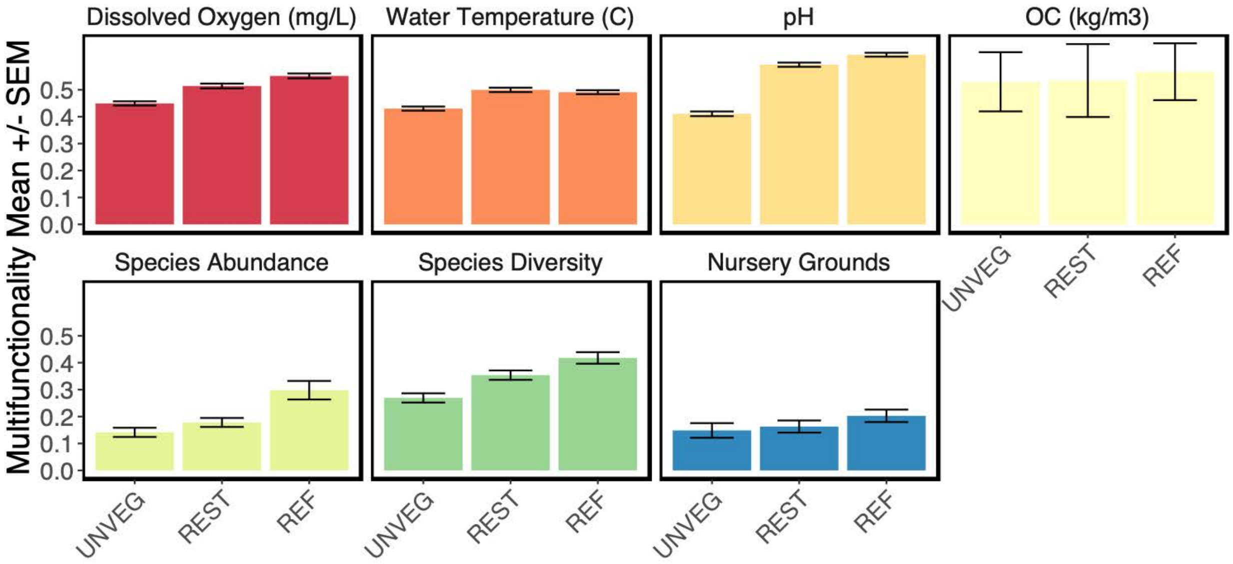

Figure S10. Average multifunctionality (± SEM) broken down by function. Top row is biogeochemical functions and the bottom row is biological functions. The y-axis is kept the same across functions to compare the relative contributions relative to the (best) 95th quantile (5th quantile for water temperature).

Appendix S1: Methods

Seagrass time series

We developed a time series using geospatial data to quantify how much new seagrass habitat in Elkhorn Slough was due to restoration plot expansion versus reference beds filling in and expanding. Specific datasets were included (or excluded) for digitization based on the clarity of the water at each seagrass site. It's possible that deeper portions of the beds closer to the channel thalweg were under-represented in our delineations due to limited visibility. However, the good consistency in the outline of the seagrass beds within each dataset group improved our confidence in the delineations. The data presented in the seagrass time series reported as Figure S1 was collected using similar aerial imagery extending as far back as 1931.

Tracking structural attributes and mesograzer abundance as a proxy for restoration survival and growth

In addition to measuring in situ canopy height, vegetative and flowering shoot density, we harvested shoots from both restored and reference plots for later lab processing of shoot, rhizome, and epiphyte biomass. We also collected data on the abundance and biomass of critical mesograzers, *Phyllaplysia taylori* and *Pentidotea resecata* from harvested shoots. While other invertebrate taxa were counted and recorded, we are only reporting on these two species because of their well-characterized role in promoting the health of seagrass beds and because they made up the majority of the mesograzer biomass across both restored and reference plots. Prior to measuring the structural attributes of harvested shoots, all invertebrates were cleared from the shoots themselves and the mesh bags they were held in.

To process each ramet, we first used a 3.8 cm single edge razor blade to scrape epiphytic algae and diatom films off of the blades and wiped them on pre-weighed labeled cotton rounds before placing them in the dehydrating oven for 72 hrs. Dry weight (g) was measured by subtracting the weight of the clean, pre-weighed cotton round from the dried cotton round with sample. Once the shoots were free of epiphytes we measured shoot lengths. Five representative shoot lengths were measured from the tip of each blade to the base of the meristem at the first (most recent) node. After shoot lengths were recorded we used the razor blade to cut the shoot from the rhizome at the most recent node. We cut each rhizome to 7 cm in length and separately wrapped each shoot and rhizome (n=50) in labeled pre-weighed foil. We poked tiny holes to allow the moisture to escape and placed the samples in the dehydrating oven for 72 hrs for biomass in dry weight (g). We then measured the dry weight of the sample by subtracting the pre-weighed foil weights from the dried sample.

Biodiversity of macrofauna

To quantify biodiversity of mobile macrofauna, a baited shrimp pot and a mid-water minnow trap was deployed in each of the habitat types. To secure the trap arrays to their desired locations and prevent them from shifting due to tidal currents, 2-4 plate weights (1.0-3.0 kg) were placed in the corners of the shrimp pots to prevent them from sliding along the seafloor and Danforth anchors were secured with line to the shrimp pots to further secure the trap arrays to their desired location. Bait (frozen anchovies or sardines) was placed in a tennis ball can with holes drilled in the base and top of the canister inside the shrimp pots. Depending on the size of the bait fish, the canisters held between 5-8 fish. Travel-toothbrush holders with holes drilled on either side served as bait containers for minnow traps and held 1 frozen fish. Mid-water minnow traps were secured to the buoy line allowing enough slack to be suspended in the water column.

Organic carbon stocks

Sediment cores were collected from October 2018-February 2020. All cores were immediately stored in a refrigerator and processed within a week. First each core was sub-sampled into 2 cm intervals and placed in a beaker to measure wet weight. Each interval was rinsed with DI water and the sediment allowed to settle over 24 hours, after which we carefully poured off the surface water and decanted the remaining water so as to prevent sediment loss. Each interval was rinsed 2-3 times (2 times for sandy sediment and 3 times for more silty sediment). Following the last salt rinse, samples were dried and the dry weight (minus salts) was recorded. Each interval was cone and quartered (Lewis and McConchie 1994) into two 10g subsamples (± 0.1000g), one for TOM analyses and the other for grain size analysis (not reported). In order to remove inorganic carbonates from the TOM subsamples, 1.2 Molar HCl was used to dissolve carbonates for 15 minutes. Afterwards, DI water was added and the sample sat for over 4 hours or until the sediment settled. The diluted acid was decanted and DI water was added again. This process of diluting the acid was repeated four times. After the final DI rinse the sediment was dried and dry weight recorded. Acid washed sediment was then subsampled using cone and quartering methods (1.3 ± 0.1000g) and placed in crucibles to burn in the muffle furnace for loss-on-ignition, LOI (Davies 1974). The muffle furnace was set to 550°C and samples burned for 3 hours. After samples cooled, crucibles were re-weighed and the difference between pre and post combustion (LOI) is the TOM that was lost. To translate TOM to organic carbon (OC), we used a power model ($y=0.22x^{1.1}$) derived using regional core data (Ward et al. 2021). Carbon storage was calculated by multiplying % OC and the bulk density of each interval, reported as kg OC m^{-3}. We used an ANOVA to determine differences in organic carbon stocks across habitats.

References: Appendix S1

Davies, B.E. 1974. Loss-on-ignition as an estimate of soil organic matter. Soil Science Society of America Journal **38**:150–151.

Lewis, D.W. and McConchie, D. 1994. Analytical Sedimentology. Boston, MA: Springer US, 197p.

Ward, M.A., Hill, T.M., Souza, C., Filipczyk, T., Ricart, A.M., Merolla, S., Capece, L.R., O'Donnell, B.C., Elsmore, K., Oechel, W.C., Beheshti, K.M., 2021. Blue carbon stocks and exchanges along the California coast. Biogeosciences **18**, 4717–4732. doi:10.5194/bg-18-4717-2021
